# Transmitted Virus Fitness and Host T Cell Responses Collectively Define Divergent Infection Outcomes in Two HIV-1 Recipients

**DOI:** 10.1371/journal.ppat.1004565

**Published:** 2015-01-08

**Authors:** Ling Yue, Katja J. Pfafferott, Joshua Baalwa, Karen Conrod, Catherine C. Dong, Cecilia Chui, Rong Rong, Daniel T. Claiborne, Jessica L. Prince, Jianming Tang, Ruy M. Ribeiro, Emmanuel Cormier, Beatrice H. Hahn, Alan S. Perelson, George M. Shaw, Etienne Karita, Jill Gilmour, Paul Goepfert, Cynthia A. Derdeyn, Susan A. Allen, Persephone Borrow, Eric Hunter

**Affiliations:** 1 Emory Vaccine Center at Yerkes National Primate Research Center, Emory University, Atlanta, Georgia, United States of America; 2 Nuffield Department of Medicine, University of Oxford, Oxford, United Kingdom; 3 Department of Medicine, University of Alabama, Birmingham, Alabama, United States of America; 4 Theoretical Biology and Biophysics, Los Alamos National Laboratory, Los Alamos, New Mexico, United States of America; 5 Human Immunology Laboratory, International AIDS Vaccine Initiative, London, United Kingdom; 6 Department of Medicine, Perelman School of Medicine, University of Pennsylvania, Philadelphia, Pennsylvania, United States of America; 7 Rwanda-Zambia HIV Research Project, Project San Francisco, Kigali, Rwanda; 8 Department of Pathology and Laboratory Medicine, Emory University, Atlanta, Georgia, United States of America; Vaccine Research Center, United States of America

## Abstract

Control of virus replication in HIV-1 infection is critical to delaying disease progression. While cellular immune responses are a key determinant of control, relatively little is known about the contribution of the infecting virus to this process. To gain insight into this interplay between virus and host in viral control, we conducted a detailed analysis of two heterosexual HIV-1 subtype A transmission pairs in which female recipients sharing three HLA class I alleles exhibited contrasting clinical outcomes: R880F controlled virus replication while R463F experienced high viral loads and rapid disease progression. Near full-length single genome amplification defined the infecting transmitted/founder (T/F) virus proteome and subsequent sequence evolution over the first year of infection for both acutely infected recipients. T/F virus replicative capacities were compared *in vitro*, while the development of the earliest cellular immune response was defined using autologous virus sequence-based peptides. The R880F T/F virus replicated significantly slower *in vitro* than that transmitted to R463F. While neutralizing antibody responses were similar in both subjects, during acute infection R880F mounted a broad T cell response, the most dominant components of which targeted epitopes from which escape was limited. In contrast, the primary HIV-specific T cell response in R463F was focused on just two epitopes, one of which rapidly escaped. This comprehensive study highlights both the importance of the contribution of the lower replication capacity of the transmitted/founder virus and an associated induction of a broad primary HIV-specific T cell response, which was not undermined by rapid epitope escape, to long-term viral control in HIV-1 infection. It underscores the importance of the earliest CD8 T cell response targeting regions of the virus proteome that cannot mutate without a high fitness cost, further emphasizing the need for vaccines that elicit a breadth of T cell responses to conserved viral epitopes.

## Author Summary

The length of time taken by HIV-1-infected individuals to develop AIDS varies widely depending on how efficiently virus replication is controlled. Although host cellular immune responses are known to play an important role in viral control, the contributions made by the infecting virus and the host antibody response to this process are less clear. To gain insight into this, we performed a detailed analysis of the interplay between the infecting virus and host immune responses in two HIV-1-infected individuals, one of whom controlled virus replication efficiently while the other did not. We found that the virus infecting the HIV-1 controller replicated much less well in culture than that infecting the progressor. The antibody responses made by both subjects were similar, but early after infection the controller mounted a T cell response targeting many sites in the virus, whilst the progressor's T cell response initially targeted only two sites, one of which rapidly mutated to avoid immune recognition. This study highlights the contribution of the replication capacity of the infecting virus and associated early induction of a broad HIV-specific T cell response, which was less readily undermined by rapid viral escape, to viral control in HIV-1 infection.

## Introduction

In the absence of antiretroviral therapy (ART) there is significant variation in the clinical outcome of HIV-1 infection [1]. Most untreated patients exhibit persistent viral replication that is detectable in plasma, and experience a gradual decline in CD4 T cells. A majority of chronically-infected, untreated individuals eventually reach CD4 T cell counts of <200 cells/µl and develop the opportunistic infections that define AIDS [2]. Some HIV-1 infected individuals progress to CD4 T cell counts of <200 cells/µl in 3–4 years (rapid progressors, [2], [3]) while a small proportion (5–15%) are slow progressors, remaining disease free for >12 years [4]–[7]. A subset of the slow progressors becomes long-term non-progressors (LTNP), remaining disease free for even longer [5], [8]. Less than 1% of HIV-1 infected individuals spontaneously control disease progression by durably suppressing plasma viral load (VL) to levels undetectable with standard assays (elite controllers (EC); VL<50 RNA copies/ml) [2], [5], [9], [10]. Recent studies on ECs have defined critical roles for host genetics, viral factors and *de novo* host immune responses in controlling disease progression [2], [8], [11], [12].

Set-point VL is considered to be a critical indicator of the trajectory for clinical disease [3], [13], [14], and we and others have recently shown that this reflects a complex interplay between the immunogenetics of the newly infected host and replication capacity of the virus, which in turn can be molded by the immune response of the transmitting partner [15]–[21]. Host immunogenetics, especially HLA class I genotype, significantly influences disease progression in the HIV-1 infected population and common genetic variants can explain about 20% of viral control [22]–[24]. The statistically significant association between protective HLA class I alleles, such as B*57, B*27 and B*81, and their additive effect on control of VL during acute and chronic infection has been shown in multiple studies [19], [21], [25]–[28]. Indeed, HLA class I-restricted HIV-specific CD8 T cell responses are the major force suppressing viremia throughout infection [5], [29], [30]. Escape mutations that are selected for in the replicating HIV quasispecies during infection can abrogate the effects of epitope-specific CD8 T cell responses [31]–[35]. However, mutations that confer escape from protective HLA alleles can also result in a high fitness cost to the virus [16], [17], [36], [37]. Moreover, in HLA-B*57+ individuals, there is evidence for continued recognition and suppression of virus with mutated epitopes [38].

The phenotype of the transmitted/founder (T/F) virus also appears to play a critical role in defining set-point VL [18], [21], [39]–[41] and disease progression [18], [42], [43]. We previously demonstrated an inverse correlation between the number of transmitted CD8 T cell escape mutations in *gag* and the set-point VL in newly-infected individuals [17]. Furthermore, transmitted HLA-associated T cell escape mutations in Gag can decrease viral replication capacity [16], [18], [20], [36]. In studies of both chronically and acutely infected EC, viruses exhibited reduced replicative capacity that was linked to T cell driven escape mutations [44]–[46]. Moreover, the Env proteins of viruses from EC utilized CD4 and CCR5 less efficiently and fused more slowly than Envs from progressing individuals [47]. Nevertheless, replication-competent virus can be recovered from some EC, suggesting that undetectable VL in these individuals is the result of immune control rather than viral defects [44], [48].

Our studies of HIV-1 transmission pairs in Zambia and Rwanda have facilitated the analysis of virus populations in both the transmitting source partner (donor partner) and the seroconverting partner (recipient partner), leading to an understanding of the impact immune selection in the donor can have on the T/F virus early in infection [18], [21], [49]–[51]. Recent technical advances allow PCR amplification and sequencing of near full-length T/F virus genomes and studies of immune selection on this initially clonal population over time [52], [53]. Moreover, knowledge of the entire T/F virus proteome permits longitudinal analysis of cellular and humoral immune responses to autologous peptides and virus [54], [55]. Use of these approaches enabled us to perform a combined analysis of the fitness of the T/F virus and viral control by host cellular and humoral responses, to better define the interplay between these viral and host determinants in determining early virus control. We studied 2 HIV-1 subtype A transmission pairs from a Rwandan cohort, in which the two recipients shared 3/6 HLA class I alleles yet exhibited distinctly different disease trajectories. One recipient rapidly progressed to a CD4 count of <350 cells/µl, while the other exhibited control of the virus. To understand the basis for differences in rate of early disease progression, we characterized near full-length viral genome sequences longitudinally, defined the *in vitro* replication capacity of the T/F virus populations, and delineated the nature and magnitude of both the autologous virus-specific T cell and neutralizing antibody (Ab) responses during the first year of infection. Results from these studies reveal that infection with a T/F virus with lower replication capacity, and the corresponding induction of a broader HIV-specific CD8 T cell response targeting more stable epitopes during primary infection were distinguishing determinants of good control of virus replication.

## Results

### Transmission pairs

Two HIV-1 subtype A male to female virologically-linked transmission pairs (R463 and R880) from a heterosexual transmission cohort at Project San Francisco in Kigali, Rwanda were studied. Both recipients were enrolled into the IAVI protocol C early infection cohort at Fiebig stage IV for R463F and Fiebig stage III for R880F (Table 1). PBMC were cryopreserved at Fiebig stage V and IV respectively. R880F and R463F shared three HLA class I alleles (B*1503, Cw*0210, and Cw*0602; Table 1). R880F also possessed A*0201, A*0301 and B*4701 alleles, while R463F possessed A*0101, A*3002 and B*4501 alleles. Interestingly, both donor partners carried one protective allele; R880M B*5703 and R463M B*8101, although only R880M exhibited a relatively low VL (approximately 14,000 copies/ml) (Table 1).

**Table 1:** Transmission pair information.

Patient ID^a^	Status^b^	Virus subtype	Fiebig stage^c^	pVL^d^ copies/ml	HLA class I alleles					
**R880M**	D	A1	NA	13,929	A*0109	A*3303	B*4415	B*5703	Cw*0407	Cw*1701
**R880F**	LR	A1	III	430,843	A*0201	A*0301	B*1503	B*4701	Cw*0210	Cw*0602
**R463M**	D	A1	NA	108,624	A*0101	A*2301	B*4415	B*8101	Cw*0407	Cw*1801
**R463F**	LR	A1	IV	152,000,000	A*0101	A*3002	B*1503	B*4501	Cw*0210	Cw*0602

a M: male; F: female.

b D: donor, LR: virologically linked recipient.

c Fiebig stage: Fiebig stage of the recipients at the time of enrollment into the IAVI protocol C early infection study, when plasma was first collected for viral sequence analysis.

d pVL: Plasma viral loads for the recipients are shown at the time of screening prior to enrollment into the IAVI protocol C early infection study, when R880F was at Fiebig stage III and R463F at Fiebig stage IV. The plasma viral load in R463F at Fiebig stage V was 3,980,000 copies/ml. Plasma viral loads in the donors are shown at the timepoint when the recipients were at Fiebig stage III/IV.

### CD4 T cell count and viral load trajectory

**Figure 1:**
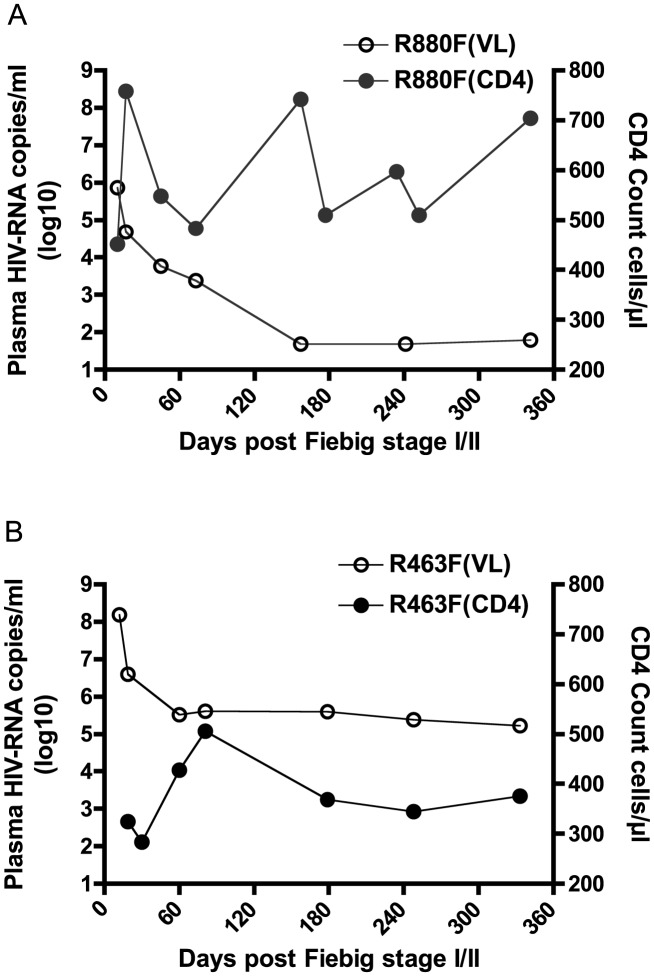
Plasma viral load and CD4 count in the two recipients during the first year of infection. Plasma viral load in RNA copies/ml is plotted on a log_10_ scale along the left vertical axis, while the CD4 T cell count in cells/µl is plotted along the right vertical axis. Time is indicated along the horizontal axis as days post-Fiebig Stage I/II in each recipient. The solid circles represent the longitudinal CD4 counts; the open circles represent plasma viral load. (A) HIV-1 elite controller R880F (B) HIV-1 rapid progressor R463F.

The highest plasma VL recorded during acute infection for R463F (VL = 152,000,000 at Fiebig stage IV) was almost 400 times higher than that for R880F (VL = 430,843 at Fiebig stage III) (Fig. 1A and 1B). Moreover, despite their similar HLA class I alleles, R880F and R463F exhibited very different VL and CD4 count trajectories during the first year of infection (Fig. 1A and 1B). By 6 months post-enrollment, subject R880F's VL had dropped to an undetectable level (VL<49 copies/ml), and remained at this level for the next 3 years of follow-up. R880F plasma from the d157 time-point was confirmed to be negative for ART. This elite controller had a CD4 count that averaged 580 cells/µl during the first year of infection (Fig. 1A). In contrast, subject R463F exhibited a more rapid disease progression. The set-point VL was around 400,000 copies/ml and the CD4 T cell count dropped to 375 cells/µl during the first year of infection (Fig. 1B). At 15 months post-infection, R463F initiated ART.

### Amplification of full-length genomes and confirmation of single virus initiation of systemic infection

Near full-length genome (NFLG; >9000 bp) viral sequences were PCR amplified from the two HIV-1 transmission pairs. These included viruses from the chronically infected donor's viral quasispecies near the time of transmission, and viruses from the recipients at 5 different time-points (enrollment, month 2 or 3, month 6, month 9, and 1 year). NFLG amplicons produced by single genome PCR amplification (SGA) from patient PBMC and plasma viral RNA were sequenced directly. At least 10 NFLG sequences from each recipient were analyzed to establish the T/F virus sequence, and neighbor-joining trees of donor and recipient virus sequences confirmed that infection was initiated by a single virus variant from the donor viral quasispecies in both cases (S1 Fig.).

### Viral isolates generated from R880F and R463F in acute infection exhibit different replication capacities *in vitro*

**Figure 2:**
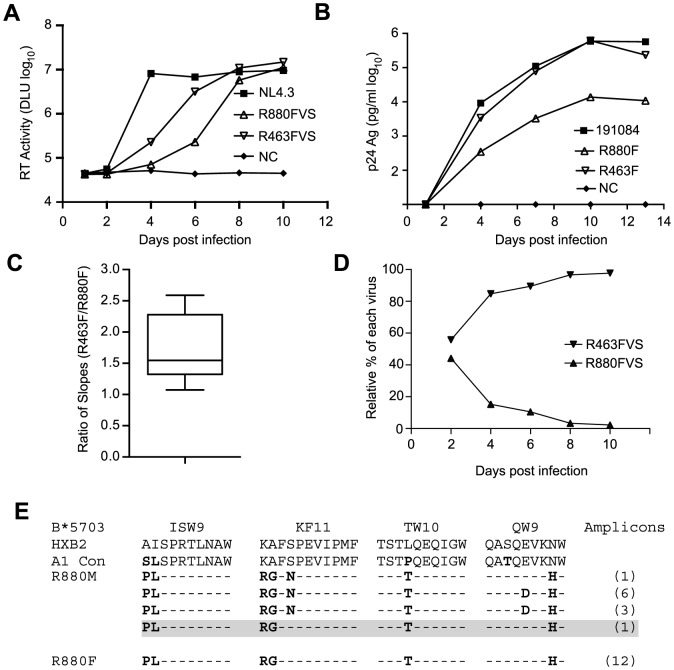
Kinetics of *in vitro* replication of viruses recovered from the earliest-available plasma samples from R880F and R463F and IMCs corresponding to each subject's deduced T/F virus sequence. (A and B). Infection with virus isolates (A) or PBMC stocks derived from IMCs (B) was performed in either pools of human CD8 depleted PBMC isolated from 3 individual donors (A) or single individuals (B), and the data is representative of at least 3 independent experiments for each. Reverse transcriptase activity (measured in digital light units (DLU) or p24 antigen is plotted on a log_10_ scale on the vertical axis against days following infection of cells in *in vitro* culture. HIV-1 NL4.3 is shown in (A) as a positive control for *in vitro* replication. (C). Analysis of the in vitro growth rates of viruses R880 and R463 from each of 9 experiments. The growth rate of each was calculated as the slope of increase in the logarithms of p24 or RT activity over the times when this increase was linear. A Wilcoxon signed-rank test was used to compare the ratios obtained in the experiments. (D). A competition growth assay was performed in triplicate as described in Methods with approximately equal input copies of R880F and R463F viral stocks. Relative amounts of each virus at days 2, 4, 6, 8, and 10 were determined by qPCR. (E). An amino acid sequence alignment of the B*5703 Gag CD8 T cell epitopes for the R880 transmission pair is shown.

The peak VL during acute infection is likely strongly influenced by the replicative fitness of the T/F virus *in vivo*. To determine whether R880F was infected with a virus with lower replication capacity (RC) than R463F, we generated virus isolates from plasma of both recipients at the enrollment time-point by growth in CD8 T cell-depleted primary PBMC from HIV-seronegative donors. The RC of these viral isolates was then compared in mixed donor, CD8 T cell-depleted, PBMC. As shown in Fig. 2A, following infection at equal multiplicities of infection, both R880F and R463F viral stocks (VS) were able to replicate; however, the R463FVS replicated more rapidly than the R880FVS (log_10_ slope 4.66/day vs 5.88/day), resulting in a delay in detectable virus production for R880FVS. To verify these observations, we also compared the *in vitro* RC of infectious molecular clones (IMCs) corresponding to the deduced T/F virus sequence from each subject (generated as previously described [56]), obtaining very similar results (Fig. 2B). We compared the relative growth of the two viruses in 9 independent experiments, and calculated the growth rates of each virus as the slope of increase in the logarithms of p24 or RT activity over the times when this increase was linear. We then used a Wilcoxon signed-rank test to compare the ratios of slopes for the two viruses with the null hypothesis that both viruses replicated at the same rate (i.e., ratio = 1). In each experiment R463 replicated faster than R880, and overall there was a statistically significant difference in the ratio of rates (Fig. 2C; p = 0.0029).

Finally, to independently confirm this difference in replicative capacity, we established a competition assay, in which CD8-depleted, activated PBMC cultures were infected with both R880F and R463F virus stocks, and the relative proportion of each virus in the culture was monitored using a qPCR-based analysis over a 10-day infection period. As can be seen in Fig. 2D and S2 Fig., whether R463FVS was in an equal ratio to R880FVS, or in a 1∶4 ratio, it rapidly outgrew the poorer replicating R880FVS. This difference in RC *in vitro* is consistent with the VL differences observed in these two acutely infected individuals *in vivo* (Fig. 1A and 1B).

R880F was infected by a virus from a partner carrying the B*5703 allele, and viral escape from CD8 T cell responses against the major B*57 epitopes in Gag has been shown to decrease virus replication [16], [57], [58]. Examination of the R880F T/F virus sequence revealed escape mutations at four B*57 CD8 T cell epitopes in p24 (ISW9, KF11, TW10 and QW9) (Fig. 2E). Surprisingly, the R880F T/F virus lacked the serine to asparagine change at position 165 in the KF11 Gag epitope that was present in the majority (10/11) of the R880M amplicons (Fig. 2E). The S165N change has been shown to compensate for the replication defect imposed by the alanine to glycine escape mutation at position 163 in KF11 found in R880M and R880F [57], [59]. Thus, lacking the KF11 compensatory change, the minor variant transmitted to R880F would be expected to have a reduced RC relative to the majority of donor variants, consistent with the *in vitro* experimental data above.

### Longitudinal analysis of virus sequence evolution over the entire proteome during the first year of infection

NFLG SGA was also utilized to investigate sequence evolution in the viral quasispecies in both R880F and R463F at four time-points up to 1 year after seroconversion. Because the VL of R880F dropped to <49 copies/ml at the 6-month time-point, sequences for the 9 and 12 month samples were derived from half-genome amplifications. A phylogenetic analysis of these sequences is shown in highlighter plots in S3 Fig., panels A and B. For R880F, despite control of VL, we observed the fixation of non-synonymous (NS) mutations in *pol*, *vif*, *rev*, *env* and *nef*, which as we show below reflect sites of selection by cellular immune responses. We observed no evidence of reversion of the transmitted T cell escape mutations in the ISW9, KF11, TW10 or QW9 epitopes during the first year of infection. In the R463F longitudinal viral sequence data, we observed fixation of NS mutations only in *gag*, *tat* and *nef*.

### Mapping of epitopes recognized by the virus-specific T cell response in subjects R880F and R463F

CD8 T cells play an important role in containment of virus replication during acute and early HIV-1 infection [34], [54], [60], [61]. Previous studies have demonstrated associations between the specificity of the CD8 T cell response to HIV-1 and containment of viral replication. Responses to more conserved proteins/epitopes, escape from which often incurs a high cost to viral fitness, are associated with better HIV control [29], [62]–[64]. To investigate whether there were differences in the specificity of the CD8 T cell responses elicited during acute and early HIV-1 infection in R880F and R463F that may have contributed to their differential infection outcomes, the epitopes in the T/F sequence recognized by autologous T cells were mapped. For each subject, a matrix of overlapping peptides corresponding to the amino acid sequence of the entire T/F virus proteome was screened in IFNγ ELISPOT assays for recognition by T cells from the 6-month time-point. Further screening was then carried out at the enrollment time-point to check for responses to sites where there was evidence of rapid selection for sequence changes during acute infection, responses to which may have declined to undetectable levels prior to the 6-month matrix screening time-point following viral escape. Having identified epitope-containing 18-mer peptides, the putative optimal epitopes recognized by T cells within these sequences and the likely presenting HLA class I alleles were predicted where possible (Table 2 and Table 3).

**Table 2:** Defined T cell targets in individual R880F.

		18mer peptide(s)			Optimal epitope	
Protein	Amino acid position^a^	Designation	Amino acid sequence	Amino acid position^a^	Designation	Amino acid sequence	HLA^b^
Gag	17–34	Gag 17–34	EKIRLRPGGKKKYRMKHL				
	81–98	Gag 81–98	TVATLYCVHQRIEVKDTK	85–95	Gag 85–95	LYCVHQRIEVK	Cw0602**
	140–157	Gag 140–157	GQMIHQPLSPRTLNAWVK				
Pol	417–434	Pol 417–434	GKLNWASQIYPGIKVKQL	424–432	Pol 424–432	QIYPGIKVK	A0301*
	425–442	Pol 425–442	IYPGIKVKQLCKLLRGTK	424–432	Pol 424–432	QIYPGIKVK	
	817–834	Pol 817–834	LKLAGRWPVKVVHTDNGS	817–826	Pol 817–826	LKLAGRWPVK	A0301**
	969–986	Pol 969–986	NSDIKVVPRRKAKIIRDY	977–986	Pol 977–986	RKAKIIRDY	B1503*
	977–994	Pol 977–994	RRKAKIIRDYGKQMAGDD	978–986	Pol 978–986		
	985–1002	Pol 985–1002	DYGKQMAGDDCVAGRQDE				
Env	350–368	Env 350–368	GEYFKNKTITFNSSSGGD				
	806–823	Env 806–823	ELKISAINLVDTIAIAVA	815–823	Env 815–823	VDTIAIAVA	B1503**
	814–831	Env 814–831	LVDTIAIAVAGWTDRIIE	815–823	Env 815–823	VDTIAIAVA	
Rev	9–26	Rev 9–26	DEELLRAIRIIKILYQSN	11–21	Rev 11–21	ELLRAIRIIKI	A0301**
Vif	25–42	Vif 25–42	VKHHMYVSKRAKRWFYRH				
Nef	177–194	Nef 177–194	EREVLKWKFDSRLALKHL	180–189	Nef 180–189	VLKWKFDSRL	A0201*
				183–191	Nef 183–191	WKFDSRLAL	B1503**

a relative to HXB2.

b presenting HLA allele previously described (*) or predicted (**).

**Table 3:** Defined T cell targets in individual R463F.

		18mer Peptides (s)			Optimal epitope		
Protein	Amino acid position^a^	Designation	Amino acid sequence	Amino acid position^a^	Designation	Amino acid sequence	HLA^b^
Gag	132–149	Gag 132–149	YPVVQNAQGQWVHQNFSP	140–147	Gag 140–147	GQWVHQNF	B1503**
	140–157	Gag 140–157	GQWVHQNFSPRTLNAWVK	140–147	Gag 140–147	GQWVHQNF	
	292–309	Gag 292–309	PFRDYVDRFFKTLRAEQA				
	381–398	Gag 381–398	GNFKGQRKIKCFNCGKE				
Pol	409–426	Pol 409–426	VNDIQKLVGKLNWASQIY	417–426	Pol 417–426	GKLNWASQIY	A3002*
	417–434	Pol 417–434	GKLNWASQIYAGIKVKQL	417–426	Pol 417–426	GKLNWASQIY	
	897–914	Pol 897–914	IHNFKRKGGIGGYSAGER				
	929–946	Pol 929–946	QKQITKIHKFRVYYRDSR	933–942	Pol 933–942	TKIHKFRVYY	B1503*
	969–986	Pol 969–986	NNDIKVVPRRKAKIIRDY	977–986	Pol 977–986	RKAKIIRDY	B1503*
	977–994	Pol 977–994	RRKAKIIRDYGKQMAGDD	978–986	Pol 978–986		
	985–1002	Pol 985–1002	DYGKQMAGDDCVAGRQDE				
Env	1–15	Env 1–15	MRVMGTQMNYQNLWRWGI				
	334–351	Env 334–351	SKAEWNETVRRVAEQLEK				
	342–360	Env 342–360	VRRVAEQLEKYFKNKTIK				
	402–420	Env 402–420	TVNATRSENDTINLPCRI				
Tat	33–50	Tat 33–50	HCLVCFQHKGLGISYGRK	38–47	Tat 38–47	FQHKGLGISY	B1503*
Nef	177–194	Nef 177–194	EGETLQWKFDSYLAFKHI	183–191	Nef 183–191	WKFDSYLAF	B1503**

a relative to HXB2.

b presenting HLA allele previously described (*) or predicted (**).

Responses were detected to a total of 12 epitope-containing regions (spanned by a single or two overlapping 18-mer peptides) in R880F (Table 2) and 13 epitope-containing regions in R463F (Table 3). As expected given the overlap in their HLA class I genotype, there were several epitope-containing regions to which responses were detected in both subjects, although around two-thirds of the regions recognized in each individual were unique. During the first 6 months of infection both subjects mounted T cell responses to multiple epitopes in the relatively conserved viral proteins Gag and Pol (7/12 of the epitope-containing regions recognized by R880F and 8/13 of those recognized by R463F), and both also exhibited 2 (R880F) or 3 (R463F) Env-specific responses, 1 Nef-specific response and 2 (R880F) or 1 (R463F) response to epitope-containing regions in other viral proteins.

### Induction of a primary HIV-specific CD8 T cell response of strikingly different breadth during acute infection in subjects R880F and R463F

**Figure 3:**
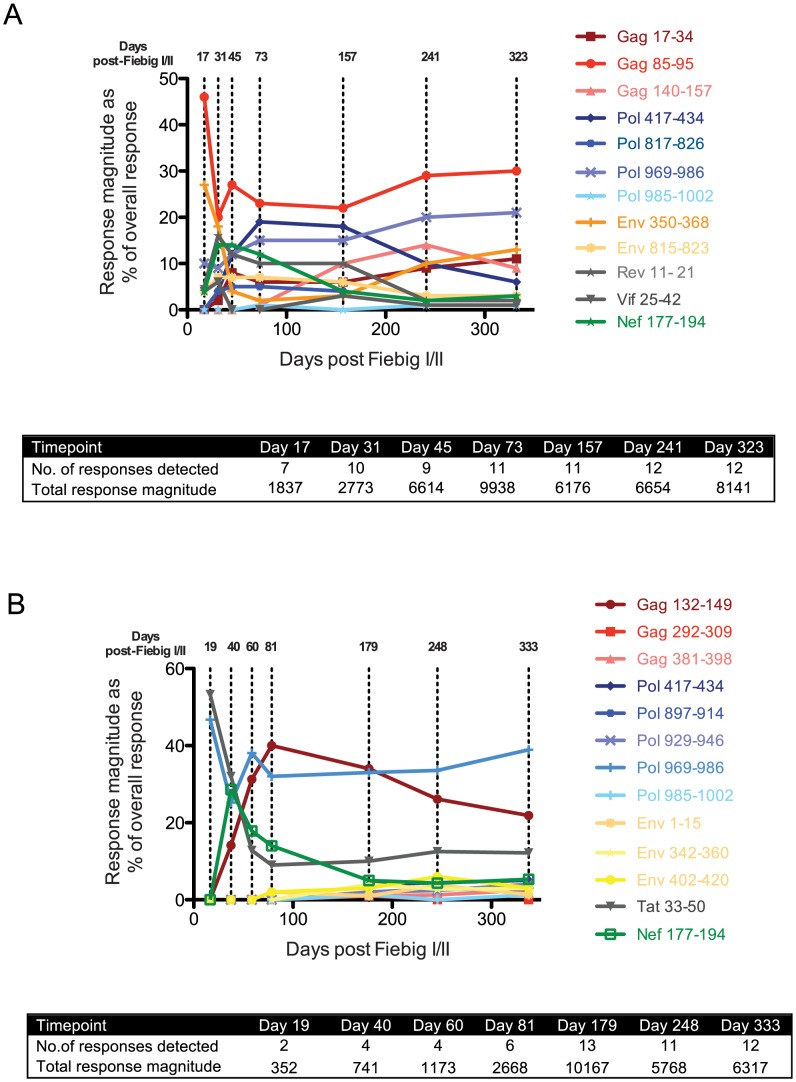
T cell response kinetics during acute and early HIV-1 infection. For both R880F (A) and R463F(B), individual peptide response magnitudes in an IFNγ ELISpot assay are shown as a percentage of the overall response. The insert shows the number of peptide responses detected and the total response magnitude (SFC/10^6^ PBMCs) at each of the time points tested. The sequences of the peptides are shown in Table 2.

Although R880F and R463F mounted T cell responses to a similar total number of epitopes in conserved and more variable HIV-1 proteins within the first 6 months of infection, we hypothesized that there may have been differences in the kinetics of induction and/or immunodominance of responses to more conserved viral epitopes during acute infection. We therefore determined the relative magnitude of responses to all the epitopes recognized in each subject (Table 2 and Table 3) at time-points over the first year of infection using IFNγ ELISpot assays (Fig. 3A and 3B). Although responses were detected to a similar number of epitopes (12–13) in both subjects at 6–12 months post-infection, there was a striking difference in their initial response breadth, with responses being detected to 7 epitopes at the earliest time-point tested in acute infection in R880F, but to only 2 epitopes in R463F. Furthermore, whereas the primary HIV-specific T cell response in R880F was dominated by a response to an epitope in Gag (Gag 85–95), with strong responses also being observed to epitopes in Pol and Env (Fig. 3A and Table 4), the initially-immunodominant response in R463F was directed against Tat (Tat 33–50), with a second response to a Pol epitope (Fig. 3B and Table 5). The initial immunodominance of a response to an epitope in Gag, combined with the greater overall breadth of the primary HIV-specific T cell response in R880F, may have limited the extent of rapid viral escape and contributed to the more effective viral control in this subject. Notably, whereas in subject R880F the initially-immunodominant Gag 85–95 response remained one of the most dominant responses throughout the first year of infection, the Tat 33–50 response that was immunodominant in primary infection in subject R463F underwent a rapid decline in relative magnitude, consistent with a selective reduction in antigenic stimulation due to viral escape (Fig. 3 and Table 5). This again suggested that there could have been differential escape from the initially-immunodominant T cell responses in these two subjects.

**Table 4:** Response and escape kinetics in individual R880F.

Epitope	Relative magnitude of epitope-specific T cell response^a^			Response avidity (uM)	Averageepitope entropy (nats)	% escape^b^				
	Day 17	Day 73	Day 157			Day 12	Day 17	Day 73	Day 157	Day 323
Gag 17–34	0%	5%	6%	6	0.4	0	0	0	0	0
Gag 85–95	46%	23%	22%	7	0.6	0	0	10	33	14
Gag 140–157	0%	1%	10%	0.8	0.2	0	0	0	0	9
Pol 417–434	0%	19%	18%	3.0	0.2	0	0	0	67	100
Pol 817–826	0%	5%	4%	1.0	0.1	0	0	0	0	0
Pol 969–986	10%	15%	15%	0.8	0.2	0	0	10	0	0
Pol 985–1002	0%	1%	0%	ND^c^	0.2	0	0	0	0	0
Env 350–368	27%	2%	3%	8	1.2	0	0	0	0	60
Env 815–823	4%	7%	6%	0.8	0.6	0	0	20	67	100
Rev 11–21	5%	10%	10%	0.9	0.8	0	0	0	67	100
Vif 25–42	4%	0%	3%	7.0	0.5	0	0	0	33	91
Nef 177–194	4%	12%	4%	6.0	0.7	0	0	100	100	100

a Relative magnitude of the epitope-specific response, expressed as % of the response detected to all epitope peptides tested, at the indicated timepoint (days post-Fiebig I/II).

b Proportion of viral genomes sequenced bearing one or more aa changes within the epitope sequence.

c ND-Not determined.

**Table 5:** Response and escape kinetics in individual R463F.

Epitope	Relative magnitude of epitope-specific T cell response^a^			Response avidity (uM)	Average epitope entropy (nats)	% escape^b^				
	Day 19	Day 81	Day 179			Day 12	Day 19	Day 60	Day 179	Day 333
Gag 132–149	0%	40%	33%	0.9	0.3	0	0	0	100	100
Gag 292–309	0%	0%	1%	ND^c^	0.1	0	0	0	0	0
Gag 381–398	0%	0%	1%	7	0.7	0	0	0	0	0
Pol 417–434	0%	0%	4%	0.5	0.2	0	0	0	0	0
Pol 897–914	0%	0%	2%	7.0	0.1	0	0	0	0	0
Pol 929–946	0%	0%	2%	2.0	0.2	0	0	0	0	0
Pol 969–986	47%	32%	32%	0.8	0.2	0	0	0	25	45
Pol 985–1002	0%	0%	1%	ND^c^	0.2	0	0	12.5	12.5	22
Env 1–15	0%	2%	1%	ND^c^	0.9	0	0	0	37	100
Env 342–360	0%	0%	4%	0.9	1.6	0	0	0	38	100
Env 402–420	0%	2%	3%	0.9	1.5	0	0	0	0	45
Tat 33–50	53%	9%	10%	7.0	0.4	10	93	100	100	100
Nef 177–194	0%	14%	5%	6.0	0.7	0	63	63	100	100

a Relative magnitude of the epitope-specific response, expressed as % of the response detected to all epitope peptides tested, at the indicated timepoint (days post-Fiebig I/II).

b Proportion of viral genomes sequenced bearing one or more aa changes within the epitope sequence.

c ND-Not determined.

### Comparison of the extent and kinetics of viral escape from the primary HIV-specific T cell response in R880F and R463F

To assess the extent and kinetics of viral escape from the T cell responses induced during acute HIV infection in the two recipients, longitudinal viral sequence evolution was monitored by NFLG SGA and sequencing (S3 Fig., panels A and B) and NS mutations occurring in the epitope-containing regions to which the patients' T cell responses were directed were identified (S1 Table and S2 Table). By 1 year post-infection, there was evidence of some degree of selection for sequence change(s) in 7/12 of the epitope-containing regions recognized in R880F and 8/13 of those recognized in R463F. Hence despite their different viral loads, extensive evolution of the viral quasispecies was able to occur in response to T cell pressure in both subjects. The impact of the amino acid changes selected for within epitope-containing regions in each subject on peptide recognition by autologous T cells was evaluated by comparing the T cell response to serial dilutions of the index and mutant peptide sequences in IFNγ ELISpot assays. In both subjects, the majority of the mutations selected for *in vivo* were found to impair or ablate peptide recognition by epitope-specific T cells (S4 Fig., panels A and B). Mutations that did not impair peptide recognition may also have conferred escape from T cell responses via effects on epitope processing (not evaluated here), suggesting that most, if not all, of the sequence changes selected for over time in epitope-containing regions represented emergence of T cell escape mutations. The observation that viral evolution to escape, at least to some extent, from multiple epitope-specific T cell responses in these two subtype A virus-infected individuals is consistent with results from previous studies in patients infected with subtype B and C viruses, which have shown that viral escape from components of the host T cell response is a hallmark of acute/early HIV-1 infection [35], [54], [62]. Notably, there was evidence of escape from only 6/15 of the T cell responses directed to epitopes in Gag and Pol, the mean entropy of which was 0.26nats, in the two recipients, whereas 10/10 of the T cell responses directed against epitopes in Env and other viral proteins, the average entropy of which was 0.89nats, showed evidence of escape by 1 year post-infection. This confirms the importance of epitope entropy as a determinant of escape from epitope-specific T cell responses during acute and early HIV-1 infection [35], [62], [65].

Although escape-conferring mutations were selected for in a proportion of the viral quasispecies by 1 year post-infection in many of the epitopes targeted by the T cell response in R880F and R463F, there were marked differences in the extent and kinetics of viral escape from the initially-immunodominant T cell responses in these subjects. In R880F, mutations did not begin to emerge in the Gag 85–95 epitope targeted by the initially-immunodominant T cell response until day 73 post Fiebig I/II. Furthermore, the mutations that occurred in the Gag 85–95 sequence did not entirely ablate epitope recognition by patient T cells, and a high proportion of the viral quasispecies retained the index epitope sequence over the entire first year of infection. This suggests that acquisition of mutations that conferred robust escape from this response incurred a high cost to viral fitness. Likewise the Env 350–368 and Pol 969–986 sequences to which strong responses were also observed during primary infection in R880F showed no evidence of sequence change until 9 months post-infection and underwent limited sequence change over the entire year of follow-up. Although mutations conferring escape from some T cell responses were selected for to completion (i.e. entirely replaced the T/F virus sequence) in this subject, the responses that were effectively escaped did not constitute major components of the overall T cell response (Table 4 and Fig. 3A). For example, the most rapidly-emerging escape occurred in the Nef 177–194 epitope, where escape variants completely replaced the index virus by day 73 post Fiebig I/II (Table 4).

By contrast, in subject R463F there was rapid emergence of effective escape mutations in the Tat 33–50 epitope recognized by the initially-immunodominant T cell response, and these mutations were represented in a majority (11/13) of the sequences at day 19 post Fiebig I/II, with selection to completion by day 60 (Table 5 and Fig. 3). The rapid escape from a high proportion of the primary HIV-specific T cell response in R463F but not R880F (S5 Fig.) likely was one of the factors contributing to the differing efficiencies of viral control in these subjects.

### Comparison of the early HIV-specific CD4 T cell response in subjects R880F and R463F

Virus-specific CD4 T cell responses play important helper roles in the immune response, and can also mediate effector activity that contributes to control of viral replication. Although CD4 T cell responses can be detected by IFNγ ELISPOT assay (and it is likely that some of the T cell responses identified in the analyses described above were mediated by CD4 T cells), many CD4 T cells do not produce IFNγ, making other approaches more suitable for CD4 T cell response analysis. We therefore employed a flow cytometry based method, where responding CD4 T cells are detected on the basis of CD154 up-regulation following antigenic stimulation (S6 Fig., panel A), to enable comparison of the relative magnitude of the Env- and Gag-specific CD4 T cell response in subjects R880F and R463F at early times post-infection. As shown in S6 Fig., panel B, R880F exhibited much higher-magnitude Gag and Env-specific CD4 T cell responses than R463F both during acute infection at ∼3 months post-infection. The more robust CD4 T cell response in subject R880F may have been consequentially and/or causally related to the lower levels of early virus replication in this subject.

### Viral Env evolution and NAb activity during the first year of infection

**Figure 4:**
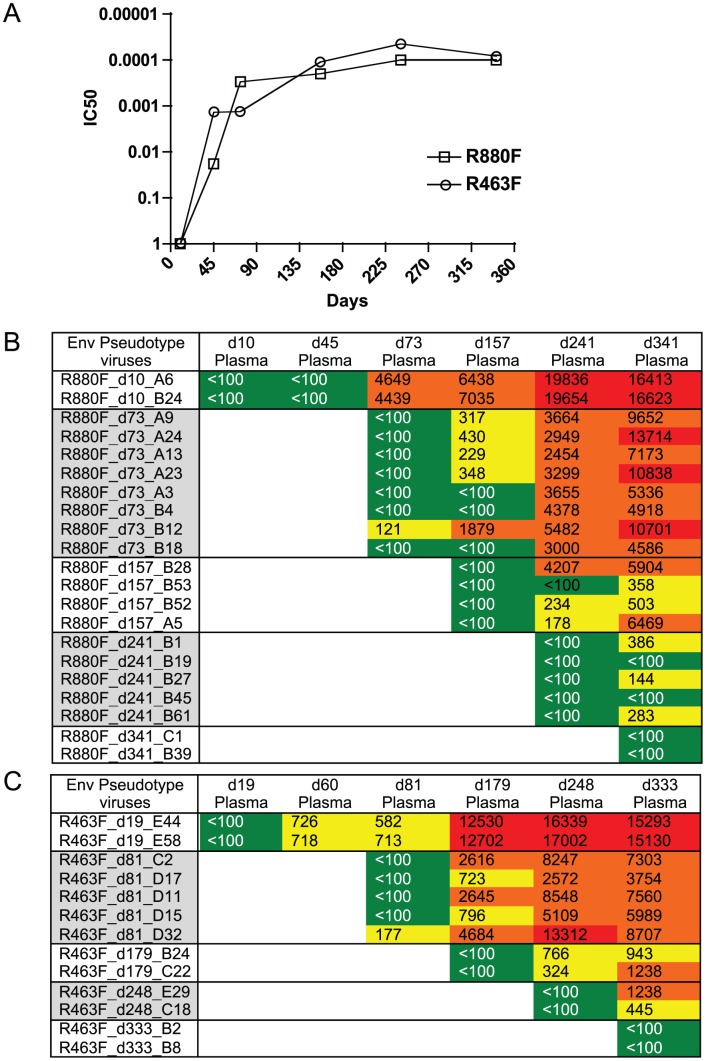
Longitudinal autologous neutralizing antibody IC50 titer during the first year of infection. (A). The IC50 neutralizing titer (plasma dilution) against the T/F virus Env-pseudotyped HIV-1 derived from either R463F or R880F is plotted on the vertical axis on a log scale. R463F is depicted by open triangles; R880F is depicted by open squares. These experiments were replicated independently at least 3 times. (B). Heat map showing autologous neutralizing Ab activity versus Envs sampled during the first year of infection. Upper Panel R880F, Lower Panel R463F. Plasma sample dates (post-Fiebig I/II) are shown across the top, and Env clones used as pseudotypes from each time point are shown on the left. Green indicates IC50 Ab titers less than 1∶100; yellow indicates IC50s between 1∶100–1∶1000; orange indicates IC50s between 1∶1000–1∶10,000; red indicates IC50s>1∶10,000.

To investigate the added selection pressure imposed by the humoral immune response, we examined the development of neutralizing Abs in both R463F and R880F over the first year of infection and correlated this with the appearance of neutralization escape mutations within the *env* sequence. To quantitate the appearance of neutralizing Ab, the *env* gene from the T/F virus was used to generate pseudotyped virions for the TZM-bl cell neutralization assay [66], [67]. Within 2 months of infection both individuals developed detectable neutralizing Ab activity against the T/F Env, and by day 73/60 robust IC50 titers of 1∶4500 (R880F) and 1∶720 (R463F) were present (Fig. 4A). At this time-point, a variety of amino acid substitutions were observed in the variable regions of gp120 (S7 Fig., panels A and B). Over the following 9 months additional amino acid changes occurred in Env gp120 of both R880F and R463F. These were found in V1, V2, V3, α2 helix, V4 and V5 regions of Env, and frequently involved changes in both the position and number of N-linked glycosylation sites (S7 Fig., panels A and B). These sequence changes were consistent with a pattern of ongoing viral escape and *de novo* neutralizing Ab production in both recipients (S7 Fig., panels A and B). Previously, the initial autologous neutralizing Ab response in R880F was mapped to an epitope at the base of the V3 domain, and early viral escape was conferred by one of three single amino acid substitutions in this region, indicated in red in S7 Fig., panel A (Murphy et al, 2013). Viral escape at later time points involved changes in N-linked glycosylation. Thus, there was ongoing selection of escape variants by neutralizing antibodies in R880F, even after the VL decreased to undetectable levels. Indeed, an analysis of neutralization of Env pseudotypes derived from d10–d341 viruses by plasma from these same time points showed that while contemporaneous viruses were resistant to neutralization, the potency against the T/F Env increased throughout the first year in R880F (Fig. 4B). Moreover, the breadth of *de novo* neutralizing antibodies in R880F continued to evolve, such that even day 341 plasma neutralized earlier Env escape variants better than plasma from previous time points (Fig. 4B). In R463F, at 1 year post-infection, when T cell depletion was evident and viral loads were very high, there was still ongoing evolution of the potency of plasma to neutralize earlier resistant variants (Fig. 4C). Nevertheless, this ongoing antibody-mediated selection pressure clearly was insufficient to contain viral load.

## Discussion

During acute HIV-1 infection viremia increases exponentially to a peak that can exceed 10^8^ RNA copies/ml (although in some subjects is substantially lower), then declines to a set-point persisting level that is a strong predictor of subsequent disease progression [3], [13]. HIV-1 specific CD8 T cell responses start to expand substantially as systemic viral spread occurs, with the earliest responses reaching peak magnitude a few days after the peak in viremia [68]–[70]. HIV-1 seroconversion is typically also detected at or just after viremia reaches a maximum [71]. Previous studies, principally focusing on subtype B and C infections, have shown that VL and its control during this early stage of infection is defined by multiple viral and host factors, including the T/F virus replication phenotype, gender, age, and class I HLA alleles of the recipient as well as host genetic markers in the donor [2], [21], [25], [37], [40], [46], [47]. Both immunological and genetic evidence indicate that viral control by CD8 T cell responses is an important determinant of set-point viremia [23], [33], [60], [61]. Importantly, however, there is limited information on how the interplay, within an individual, between viral replicative fitness and host humoral and T cell responses might determine viral control. In this study, we therefore investigated the basis of immune control during the earliest stages of infection by analyzing, from both a virological and immunological standpoint, two transmission pairs with distinct disease trajectories in a Rwandan cohort infected by viruses from the relatively understudied HIV-1 subtype A.

The HIV-1 transmission pairs studied were identified sufficiently close to the time of transmission that, using single genome near-full length PCR amplification, we could determine the sequence of the single T/F virus that infected each recipient and define immune-driven evolution across the entire proteome in the context of the HLA class I profile of both recipient and donor. Moreover, we could compare the T/F sequence to that of a sampling of the donor quasispecies near to the time of infection, confirming the genetic bottleneck that occurred during transmission. The two extremes of HIV-1 pathogenesis observed for these newly infected partners (R880F and R463F), developed despite the fact that they shared 3/6 HLA-I alleles. This allowed us to probe how the properties of the infecting virus and early immune responses involved in virus control might contribute to the two different outcomes.

Replication competent virus stocks, derived from Fiebig stage III/IV plasma, and T/F IMCs for both recipients were used in *in vitro* replication assays in PBMCs, which showed that the virus infecting R880F had a significantly lower RC than that derived from R463F and that the latter rapidly outcompeted R880F in competition assays, consistent with the >100-fold difference in plasma VLs at Fiebig III/IV stage of infection. A likely basis for this difference was defined by an analysis of the virus in transmitting partner of R880F, who carried the favorable HLA class I allele B*5703. The *gag* sequence of the T/F virus contained mutations consistent with immune escape in all 4 of the previously described B*5703 Gag epitopes. Interestingly, a majority (10/11) of the R880M viral genomes encoded both the A163G and the compensating S165N mutation [57], [59] in the KF11 Gag epitope. This compensatory mutation was absent in one R880M genomic sequence and, consistent with the reduced RC observed for the T/F virus, in the T/F virus sequence of R880F. Moreover, there was no reversion of any of the B*5703 Gag epitope escape mutations during the first year of infection. Thus it appears that a less abundant, replication defective virus was transmitted from R880M to his partner, which likely contributed to her lower Fiebig III VL. In contrast, the T/F virus from R463F, in whom the maximum-recorded VL was 1.52×10^8^ HIV-1 RNA copies/ml, replicated efficiently *in vitro*. In an analysis of the *in vitro* RC of chimeric viruses with *gag* genes derived from 150 acutely infecting subtype C viruses, we showed that both lower VL and slower CD4 decline were significantly associated with low RC viruses [18]. Moreover, lower fitness viruses have been shown to be transmitted from individuals carrying protective HLA alleles, and have also been associated with elite control of VL [2], [15], [17], [18], [46], [72], [73]. Thus the difference in viral RC and peak VL between R880F and R463F may be a key contributor to the different disease trajectories in these subjects.

Use of near full-length SGA to investigate longitudinal viral sequences from both recipients also allowed us to define viral genomic evolution, driven by the *de novo* immune response, in the linked recipient over the first year of infection. Surprisingly, during this period, we observed evidence for selection of mutations at only a limited number of positions. For the controller, R880F, outside of Env, where we showed early selection of multiple neutralization escape mutations and evidence for T cell-driven escape at two sites, fixation of amino acid changes was observed in Pol, Rev, Tat and Nef. In each case the sites of mutation corresponded to epitopes recognized by the subject's T cell response. It is of interest that for the most part, selection and fixation of these T cell escape mutations in R880F occurred after VL had been suppressed to <49 [74] copies/ml. This indicates that despite viremia being below detectable levels, sufficient virus replication was nonetheless occurring for escape mutations to be generated and, in the context of pressure from the T cell response, to reach fixation in the quasispecies. For the progressor, R463F, even fewer residues showed evidence of immune escape outside of Env, where, in addition to escape from neutralization, two residues at sites targeted by T cells also escaped. Fixation of amino acid changes occurred in Gag, Tat and Nef, which again correlated with detection of T cell responses to these sites. The fixation of mutations at fewer sites in the quasispecies during early infection in R463F than in R880F, despite the much higher levels of ongoing virus replication in the former subject, suggests that the T cell responses in subject R463F exerted less pressure on viral replication than those in R880F, likely as a result of a decline in their antiviral efficacy, which both we and others have shown occurs during early infection in subjects who do not control viremia efficiently [75]–[77].

We were able to use the sequence of the T/F virus in each recipient to construct matrices of overlapping peptides corresponding to the entire autologous T/F virus proteome, which enabled comprehensive analysis of the epitopes recognized by the T cell responses induced in each subject during the first 6 months of infection. The kinetics of the response to each epitope were also analyzed. For R880F, a broad T cell response was observed even at the earliest time of sampling, with 7 epitopes in Gag, Pol, Env (2), Rev, Vif and Nef being recognized by the primary T cell response. The most dominant responses at this stage were to epitopes in Gag (Gag 85–95) and Env (Env 350–368). The former was predicted to be restricted by HLA-Cw0602, an HLA-C allele with relatively high expression levels, a phenotype recently reported to be associated with good HIV-1 control, putatively as a consequence of restriction of efficacious HIV-specific T cell responses [78]. There was no evidence of rapid emergence of escape mutations at either of these sites. Furthermore, although some mutations were observed in the Gag epitope by ∼3 months post-infection they were not strongly selected for over time, and remained a low proportion of the quasispecies even at one year post-infection (14%). This suggests that acquisition of escape mutations in/around this epitope in the Gag matrix domain might result in an unacceptable loss of viral replicative fitness. Similarly, the site targeted in Env (350–368) is very close to residues involved in the CD4 binding site, which is critical to virus infectivity.

The primary T cell response in R463F was quite distinct, being much narrower and targeting only two epitopes (Pol 969–986 and Tat 33–50) at the earliest time-point tested. The more immunodominant of these responses was directed against the Tat epitope, which by day 12 post Fiebig I/II was already exhibiting evidence of escape with 10% of the sequences mutated from that of the T/F, and more than 90% escape on day 19. Thus, the earliest CD8 T response in this individual was predominantly targeted at an epitope that was easily mutated with presumably little replicative fitness cost. Interestingly, although this Tat epitope was presented by HLA B*1503 and thus might have been expected to be a common response in both recipients, it was not detectably targeted by R880F, likely because existing mutations in this peptide (FLNKGLGISY vs FQHKGLGISY) abrogated HLA binding and precluded recognition. Thus, the acquisition by R880F of a T/F virus with a mutated Tat protein may have spared early targeting of an easily escaped epitope. The initially narrow response in R463F broadened over the next few months such that by 6 months post-infection a total of 13 epitopes were recognized, with dominant responses to epitopes in Gag (Gag 132–149) and the original Pol epitope, but despite this VL remained >300,000 copies/ml. Notably, even when the response increased in breadth, R463F did not recognize the Gag 85–95 epitope targeted by the most immunodominant T cell response in R880F despite the fact that, like the Tat 33–50 epitope, this was predicted to be presented by a HLA allele shared by both recipients. Again, the viruses transmitted to the two recipients showed sequence differences within and around this epitope (LYCVHQRIEVK in R880F and LYCVHRKIDVK in R463F) that may have precluded epitope presentation and/or recognition in R463F. These observations underline the importance of the T/F virus sequence in shaping the specificity of the host T cell response: despite the overlap in their class I genotypes, the initially-immunodominant T cell responses in R880F and R463F were entirely different, with R463F targeting a rapidly-mutating Tat epitope that was not conserved in R880F's T/F virus sequence and a Pol epitope that was recognized by R880F, but was subdominant in R880F's response to a low entropy Gag epitope that was not conserved in R463F's T/F virus sequence.

Previous studies [79] have failed to observe an association between the breadth of the CTL response in early chronic infection and set-point VL, and if we consider responses at 3–6 months post-infection, that is also the case here. However, this is not so at acute time-points (days 17 and 19 post Fiebig I/II) when major differences in the breadth of the response were observed in these two subjects who exhibited disparate abilities to control virus replication. The limited epitope breadth of the primary HIV-specific T cell response in R463F is not unusual: we and others have found that initial expansion of responses to only a limited number of viral epitopes, followed by delayed expansion of additional responses, occurs in a high proportion of HIV-1-seroconverting subjects [62], [70]. In a previous study where we conducted a proteome-wide analysis of the kinetics of epitope-specific T cell response expansion in 21 subtype B acutely-infected individuals, responses were observed to a median of only 2 epitopes at the earliest time-point tested (between 5 and 32 days following onset of symptoms, which typically develop a few days after the Fiebig I/II transition) [70]. In these subjects, we did not observe a correlation between the response breadth at the earliest time-point tested and the subsequent set-point VL. This may have been because many of the subjects in this cohort established moderate-high persisting viral loads or because not all subjects studied were sampled sufficiently early to enable accurate assessment of the initial response breadth; and/or may indicate that response breadth is not itself the most critical determinant of the efficiency of viral control. Instead, the extent and kinetics of viral escape from the earliest epitope-specific CD8 responses may be the key determinant of the efficiency of early HIV-1 control, with response breadth influencing viral control indirectly via its effect on the efficiency of escape [80], [81].

The primary HIV-specific T cell response in R880F was not only considerably broader than that in R463F, but its most dominant components also targeted epitopes in sites where acquisition of escape-conferring mutations was likely to incur significant costs to viral fitness, and where escape mutations emerged with delayed kinetics and were not selected for to completion in the viral quasispecies during the first year of infection. By contrast, the more dominant of the two responses initially induced in R463F targeted a rapidly-mutating epitope in Tat, which was already mutated in the transmitted founder virus in R880F in whom no T-cell response to it was detected. Thus, in this subject, the immune system was not “distracted” by an epitope that could rapidly escape. This may have been a critical component of control, since during acute SIV infection escape from immunodominant Mamu-B*0801-restricted CD8 T cell responses was found to differentiate Mamu-B*0801+ macaques that subsequently underwent rapid disease progression from those controlling viral replication [82]. Moreover, the association of HLA-B*2705 with good HIV-1 control is thought to be largely attributable to early targeting of a highly conserved Gag epitope by the most immunodominant HLA-B*2705-restricted T cell response, escape from which is typically not observed until many years post-infection [31]. There is also strong evidence that early targeting of responses to conserved epitopes where escape can occur, but only at a high cost to viral fitness, is associated with good HIV-1 control [26], [29], [83]. As epitope entropy is an important determinant of the rate of emergence of escape mutations during acute HIV-1 infection [62], [65], the beneficial effect of targeting conserved epitopes may be due in part to the delayed kinetics with which the most immunodominant components of the primary T cell response are escaped in acute infection.

Both R463F and R880F exhibited responses to a similar number of epitopes by ∼6 months post-infection. As both subjects were capable of mounting T cell responses to a similar number of viral epitopes, it is unclear why the initial T cell response in R463F was much narrower than that in R880F. The difference observed in the magnitude of the HIV-specific CD4 T cell response in these subjects is unlikely to explain this, as the priming and initial expansion of CD8 T cells does not require help from CD4 T cells [74]. Instead, it is more likely to be have been due to differences in dendritic cell (DC)-mediated T cell priming. We have found that apoptotic microparticles, which reach high concentrations in the circulation during acute HIV-1 infection as extensive destruction of CD4 T cells occurs [84], inhibit DC functions, impairing the ability of DCs to produce IL-12 and prime T cell responses [85]. As the magnitude and dynamics of the increase in circulating levels of apoptotic microparticles parallel those of viremia, DC functions may be rapidly and severely impaired in patients such as R463F who experience a high-magnitude acute viremic burst, resulting in initial priming of T cell responses to relatively few epitopes, with responses of additional specificities being primed only after viremia declines and DCs are replenished. By contrast, in a patient such as R880F, infected with a less fit virus, and where peak viremia is lower, DC functions may be less severely compromised and/or a greater breadth of T cell responses may be initiated prior to impairment of DC functions, resulting in expansion of a primary T cell response of greater epitope breadth. The fitness of the transmitted virus may therefore impact on set-point viremia both directly and also indirectly, by influencing the breadth of the primary HIV-specific CD8 T cell response and subsequent efficiency of T cell control of viremia.

The role of neutralizing Ab (NAb) in elite control at early phases of infection is not understood, and how it might influence the control of HIV-1 infection and disease progression remains controversial [86]. Early studies focused on HIV-1 LTNP showed that in some cases increased NAb breadth was associated with reduced disease progression [4], [87]–[89], while in other studies NAb were found to be lower in the EC/LTNP subgroup in comparison to chronic progressors [90]–[93]. In this study, we observed a robust autologous NAb response to the T/F virus in both R880F and R463F, and a more detailed analysis of the development of the Ab response in R880F demonstrated multiple pathways of viral escape followed by *de novo* neutralization [94]. Previous studies have demonstrated that early NAb escape involves single amino acid substitutions, insertion and deletion of amino acids in the variable loops of gp120, and shifts in the positions of glycosylation sites in gp120 [94]–[104]. In the current study, we observed no significant differences in the magnitude or kinetics of autologous NAb during the first year infection of R880F and R463F. In the former, two single mutations identified at day 73 post Fiebig I/II, one just upstream of the V3 loop (I295R) and one in the α2Helix (E338K) regions, were responsible for the earliest NAb escape, and pinpointed the first NAb epitope targeted in this individual [94]. Fixation of neutralization escape mutations provides evidence for ongoing selective pressure on the virus with replacement of a susceptible population by one resistant to neutralization, and this was observed in the α2 helix region of R880F at day 157. Glycan shifts and additions were also observed in R880F that also conferred escape at later time points. Fixation of new mutations longitudinally in Env and an evolving Ab response even after VL is below detection is further evidence of ongoing virus replication and continued pressure on the virus that presumably could contribute to continued immunological containment.

In R463F early NAb responses appeared to be focused on the V4 region of Env, rather than the α2-helix, with half of the amplicons (9/18) at this time-point exhibiting the addition of a glycosylation site in this region, which was associated with increased virus neutralization resistance. In contrast to R880F, a more diverse population of Envs developed over time with deletion and insertion mutations in V4 and V1 observed at the d179 and d248 time points. The selection of these more drastic mutations may reflect the higher RC of the virus and the generation of more diverse errors, consistent with lack of control of VL.

Together, the results from this study highlight the combined roles of the fitness of the T/F virus and the efficiency of viral control by host T cell responses in determining the outcome of HIV-1 infection. Whilst the relative contributions of the two cannot be precisely determined, our findings suggest that the replicative capacity of the transmitted virus may be particularly important. T/F virus fitness may not only play a direct role in determining the magnitude of the acute viral burst and subsequent levels of viral replication, but may also have a simultaneous impact on the efficiency of host immune control of viral replication, influencing early T cell response breadth and hence patterns of viral escape, and dictating the availability of CD4 T cell help to sustain T cell effector functions over time. In line with this, it is notable that genetic studies have found that only ∼22% of the variation in set point viremia can be explained by common polymorphisms in/around the MHC class I locus [23], [24]. Whilst this may reflect an important role for rare host genetic variants in dictating levels of early virus replication, it is tempting to speculate that the nature of the infecting virus may be a key determinant of HIV-1 infection outcome.

For HIV-1 vaccine development, understanding the complex interactions between virus and host that result in HIV-1 elite control at the earliest phase of infection is crucial. This study has provided a unique opportunity to investigate in a comparative fashion the viral phenotype and host adaptive immune responses in two individuals with highly disparate disease outcomes during the earliest phase of infection. Overall these results suggest that in the rapid progressor, infection by a robustly replicating virus, coupled with a narrow T cell response that was in part focused on a rapidly mutating epitope precluded viral control. In contrast, in the elite controller, a combination of infection by a less replication competent virus, which may have spared the CD4 repertoire, and a diverse T cell response to epitopes that remained stable over the first few months of infection, implying subsequent escape may have incurred a higher cost to viral fitness, allowed near complete suppression of virus replication. It will be important to extend similar, comprehensive studies to a larger number of infected individuals in future, to determine the generality of the observations reported here. However, these findings highlight the importance of early viral replication in determining subsequent viral control, emphasizing the need for vaccines to elicit rapidly acting responses that will constrain virus replication in the earliest stages of infection. They also underscore the importance of the earliest CD8 T cell response targeting regions of the virus proteome that cannot mutate without a high fitness cost, further emphasizing the need for the development of vaccines that elicit a breadth of T cell responses to conserved viral epitopes.

## Methods

### Ethics statement

This study was approved by the Rwanda National Ethics Committee in Kigali, Rwanda and by the Emory University Institutional Review Board. All individuals enrolled into this cohort provided written informed consent.

### Study subjects

The two HIV-1 subtype A transmission pairs investigated in this study were enrolled in the heterosexual discordant couple cohort at Projet San Francisco in Kigali, Rwanda. The HIV-1 serodiscordant couples received counseling and testing on a monthly basis prior to the negative partner becoming HIV-1 positive. The recipients were enrolled in the International AIDS Vaccine Initiative (IAVI) Protocol C early-infection cohort. The epidemiological linkage status for each transmission pair was defined by phylogenetic analyses of HIV-1 *gp41* sequences between cohabiting partners [105]. Both recipients were ART naïve during this study period. Infection in R880F was detected at Fiebig stage III, when plasma samples were collected; and PBMC were first cryopreserved 7 days later following seroconversion to Fiebig stage IV. Infection in R463F was detected at Fiebig stage IV, when plasma samples were collected; PBMC were first cryopreserved 7 days later at Fiebig stage V. Days post Fiebig stage I to stage II were calculated for each sample time-point based on previously reported time intervals [71].

### Plasma viral RNA/genomic DNA extraction and cDNA synthesis

Plasma viral RNA and genomic DNA were isolated using the QIAamp RNA and DNA mini kits (Qiagen, Valencia, CA), and cDNA synthesis was carried out using Superscript III (Invitrogen). For plasma samples with VL<50 copies/ml, 1 ml of plasma was ultracentrifuged for 2 h at 114,000 g, and the viral pellet was resuspended in 140 µl of residual plasma before the extraction. Reverse transcription of extracted RNA was carried out in two stages: in the 1^st^ stage incubation was at 50°C with 5 U of reverse transcriptase (RT) for 1 hour with 0.5 mM of each dNTP, 5 mM DTT, 2 U/µl RNaseOUT (RNase inhibitor), and 0.25 mM antisense primer; the 2^nd^ stage involved incubation at 55°C with an additional 5 U of RT for 2 hours. Synthesis was initiated by reverse primer: 5′-ACTACTTAGAGCACTCAAGGCAAGCTTTATTG-3′ [52], and terminated by incubating at 70°C for 15 min, followed by 20 min at 37°C with 1 µl RNase H. The cDNA was used immediately for PCR amplification.

### Near full-length HIV-1 genome amplification

For single genome PCR amplification, the cDNA was serially diluted and the dilution yielding approximately 30% NFLG PCR positive reactions was used [51], [106] to ensure a majority of the amplicons were derived from a single viral cDNA molecule. The first round PCR primers used for this nested PCR reactions were:

sense primer 1.U5Cc - HXB2 positions 538–571 –

5′-CCTTGAGTGCTCTAAGTAGTGTGTGCCCGTCTGT-3′,

and antisense primer 1.3′3′PlCb at HXB2 positions 9611–9642 –

5′-ACTACTTAGAGCACTCAAGGCAAGCTTTATTG-3′;

2^nd^ round primers were:

sense primer 2.U5Cd at HXB2 positions 552–581

5′-AGTAGTGTGTGCCCGTCTGTTGTGTGACTC-3′

and antisense primer 2.3′3′plCb at HXB2 positions 9604–9636 –

5′-TAGAGCACTCAAGGCAAGCTTTATTGAGG-3′ [52].

PCR reactions were carried out by using the Expand Long Template PCR System, (Roche, cat. no. 11 681 842 001) as recommended by the manufacturer with 0.35 µM dNTP (10 mM each), 0.2 µm sense and antisense primers and 2 U of Expand Long Template DNA polymerase. After an initial 2 min at 94°C, the first 10 cycles were 94°C 15 sec denaturation, 60°C 30 sec annealing and 68°C 9 min extension; followed by 20 cycles of 94°C 15 sec, 60°C 30 sec and 60°C 9 minutes+20 sec/cycle for the extension step. The final PCR product was incubated at 72°C for 20 minutes prior storage at 4°C. All the procedures were performed under strict clean-room procedures to prevent contamination.

### Sequencing and viral sequence analysis

The entire ∼9 kb PCR fragments were sequenced using cycle-sequencing with BigDye terminator chemistry and protocols provided by Applied Biosystems and then analyzed using an ABI 3730xl genetic analyzer (Applied Biosystems) at the University of Alabama at Birmingham Sequencing core. All sequences were aligned using the Gene Cutter tool (Los Alamos National Laboratory - http://www.hiv.lanl.gov/content/sequence/GENE_CUTTER/cutter.html). Sequences were analyzed phylogenetically using Geneious software (Biomatters, Aukland, NZ) and the highlighter tool (http://www.hiv.lanl.gov/content/sequence/HIGHLIGHT/highlighter_top.html). Entropy analyses were performed using the LANL Entropy tool (http://www.hiv.lanl.gov/content/sequence/ENTROPY/entropy.html). These analyses allowed us to define the T/F virus sequence and to visualize sequence polymorphisms and evolution in longitudinal sequence data in order to assess potential T cell and neutralizing Ab escape mutations and compensatory mutations.

### Acute infection virus isolation and T/F IMC generation

Virus stocks were isolated from p24 positive patient plasma cryopreserved from each subject at the earliest available timepoint in infection (Fiebig stage III (R880F) or IV (R463F)). A total of ∼50×10^6^ PBMCs from a mix of 3 HIV-seronegative donors were used to generate CD8-depleted pools of activated PBMC. Cells were stimulated by incubation for 72 hours at 37°C in 5% CO_2_ in RPMI medium with 20% FBS containing 20 U/ml IL-2, 50 ng/ml OKT3 anti-CD3 Ab (R&D Systems, Minneapolis, MN) and 100 ng/ml anti-CD28 Ab (eBioscience, San Diego, CA). CD8 depletion was performed using MACS LD columns (MIltenyi Biotec) as per protocol. A total of 1 ml plasma was mixed with 50 µl anti-CD44 beads (MACS molecular), and incubated at 4°C for 30 minutes with agitation. The CD44/virus combination was mixed with the CD8-depleted PBMC (20×10^6^ cells) in 4 ml of RPMI containing 10% FBS and spinoculated at 25°C for 2 h at 1200 g. The cell pellet was resuspended in 15 ml RPMI with 20% FBS and 20 U/ml IL-2 and incubated for 48 hrs at 37°C, 5% CO2. The infection culture was expanded by spinoculating the initially infected cells with an additional 20×10^6^ cells for 2 hours at 1200 g. Virus was harvested sequentially on days 5, 7, 9, 11 and 13 and titrated on TZM-bl cells as described previously [50], [66]. To confirm the identity of the virus stocks, 5 NFLG were PCR amplified from d9 stocks and sequenced from both. The consensus sequence derived from these was confirmed to be identical to that of the NFLG amplified at these time-points from the patient plasma. IMCs corresponding to the deduced T/F virus sequence of each recipient were generated and infectious virus was recovered by transfection of 293T cells as previously described [56]. PBMC-derived virus stocks were then generated from the 293T cell supernatants.

### *In vitro* replication capacity assay

For studies with acute virus derived stocks, a total of 5×10^5^ CD8 depleted PBMCs were infected at an MOI of 0.01 in a 96-well plate, in triplicate. The plate was incubated at 37°C for 3 hours, then centrifuged at 2000 RPM for 5 minutes. A total of 150 µl of the media was removed and replaced with fresh RPMI with 10% FBS to remove unbound virus, and this wash step was repeated 4 times. The resuspended cell pellets were then transferred to 24-well plates, with 1 ml RPMI with 15% FBS, 20 U/ml IL-2. The plate was incubated at 37°C, 5% CO2, and 0.5 ml supernatant was harvested and replaced with fresh medium on days 0, 2, 4, 6, and 10. Virus spread through the culture was quantitated using a reverse transcriptase assay as described recently [18].

For studies of IMC derived virus, 293T cells were transfected as described previously [56], and the resulting virus was added to 0.5 million CD4 T cells derived from individual donors at various multiplicities of infection (0.02, 0.1, 0.5). After a 2 hour incubation the cells were washed 3 times, then virus production was assessed by removing 0.06 ml culture medium at the indicated days post-infection (culture medium was not replenished) and quantitating p24 levels.

### Competition replication assay

For the competition replication assay, input virus concentrations were adjusted to have equal and 1∶4 ratios of R463 and R880 RNA copies as assessed using the qPCR quantitation described below. Anti-CD3/anti-CD28 activated, CD8+ T cell-depleted, PBMCs were infected at a total MOI of 0.01 in triplicate as described above but in 24-well plates, and 0.5 ml samples were removed on days 2, 4, 6, 8 and 10. RNA was extracted, using Qiagen mini viral RNA columns (Qiagen, Valencia, CA) from 140 µl of clarified culture supernatant for each sample and 1/5 of the eluted RNA was used for cDNA synthesis, using superscript III, as described by the manufacturer. Samples were diluted in nuclease-free water and analyzed simultaneously by quantitative real-time PCR (qPCR) for each virus.

HIV Clade A integrase primer and probe sequences are:

Fwd 5′-GTTATYCCAGCAGARACAGG-3′,

Rev 5′-TGACTTTGRGGATTGTAGGG-3′,

R880 probe 5′-GCCTGTTGGTGGGCCGGC-3′.

R463 probe 5′-GCCTGTTGGTGGGCAAAT-3′.

qPCRs were performed using the Taqman Universal master mix (Life Technologies), 0.2 µM of each primer, and 0.125 µM of probe. All assays were performed on the ABI 7500 systems (Life Technologies). RNA copy numbers were calculated from standard curves derived from known concentrations of linearized plasmids encoding the respective integrase region, and relative proportions of each virus calculated and graphed.

### Statistical analysis

To compare the in vitro growth rates of viruses R880 and R463, the data from each of 9 experiments was used to calculate the ratio of the growth rates of R463 to R880. The growth rate of each was calculated as the slope of increase in the logarithms of p24 or RT activity over the times when this increase was linear. By calculating the ratios, which lack units, the data from both types of experiment can be used together. The assumption was made that experiments with different MOI and/or different donor PBMC provide independent estimates of the growth rates. A Wilcoxon signed-rank test was used to compare the ratios obtained in the experiments, with the null hypothesis that both viruses replicated at the same rate, i.e. this ratio is 1.

### Synthetic peptides

18-mer peptides overlapping by 10 amino acids were synthesized (Sigma- Aldrich, UK) to match the sequence of the entire proteome of the founder viruses from R880F and R463F. Approximately 400 sequence-matched peptides for each patient were arranged into 126 pools in a matrix format using the Peptide Portal program (Statistical Center for HIV/AIDS Research and Prevention, US) adapting code from the Deconvolute this! program [107]. Each peptide was repeated three times in the matrix peptide plate.

For the fine-mapping of responses towards overlapping 18mers, shorter peptides spanning previously described HLA-matched optimal CD8 T cell epitopes as listed on the LANL database (http://www.hiv.lanl.gov/content/index) and predicted CD8 T cell epitopes using the syfpeithi database (http://www.syfpeithi.de/) and/or NetMHC 3.2 Server (http://www.cbs.dtu.dk/services/NetMHC/) were generated (Sigma- Aldrich, UK).

### Mapping of the HIV-specific T cell response

HIV-specific T cell responses were mapped by IFNγ ELISpot assay using a peptide matrix screening approach as described previously [70]. Briefly, cryopreserved patient PBMC from 6 months after enrollment were tested by IFNγ ELISpot assay (Mabtech, Sweden) for responses to a matrix of peptide pools (each pool containing up to 10 subject-specific peptides, each at a final concentration of 10^−5^ M) corresponding to the autologous T/F virus sequence. From the matrix screening a ranked list of potential epitope-containing peptides was deduced using the Peptide Portal program (Statistical Center for HIV/AIDS Research and Prevention, US) adapting code from the Deconvolute this! program [108]. Putative epitope-containing peptides were retested individually at 10^−5^ M in second round assays using PBMC from 3 and 9 months after enrollment. Peptides stimulating responses measuring >3× background counts and >50 IFNγ spot-forming cells per million PBMC were considered positive in these assays. To enable identification of any responses that were rapidly escaped during acute infection and had declined in magnitude to below the assay cut-off by the 6-month matrix screening time-point, peptides spanning all sites where rapid selection for sequence change was observed were also screened for recognition by PBMC from the enrollment time-point. Where possible further fine-mapping of optimal epitopes within responding overlapping 18mers were performed using smaller peptides spanning known or predicted HLA class I-restricted epitopes (final concentration 10^−5^ M).

### Measurement of the functional avidity of T cell responses

The functional avidity of T cell responses at sequential time-points during the first year of HIV infection was determined by peptide-titrated IFNγ ELISpot assay. Index sequence (autologous T/F sequence) peptides were titrated (either as responding 18mer and/or fine-mapped optimal peptide) at final concentrations ranging from 10^−5^ M and 10^−8^ M in duplicate against a constant number of PBMC (1.5–2×10^5^/well). The functional avidity was determined as the peptide concentration required to elicit half of the maximal IFNγ response in the assay.

### Analysis of effect of intra-epitopic sequence variation on T cell recognition (escape analysis)

Patient PBMC (1.5–2×10^5^/well) were stimulated with log-fold titrations of either index or variant sequence peptide(s) in IFNγ ELISpot assays. A variant peptide was deemed an escape variant if its half-maximal stimulatory concentration was at least 10-fold higher than that of the index sequence peptide. Average epitope entropy calculations for the CD8 T cell targets were based on HIV subtype A, B and C sequences listed on the LANL database (http://www.hiv.lanl.gov/content/index) and, where available, published subtype A sequences [109]. Calculations were performed as previously described [62].

### Analysis of HIV-specific CD4 T cell responses

HIV-specific CD4 T cell responses were analyzed by measuring the proportion of CD4 T cells up-regulating CD154 in response to stimulation with viral peptide pools using flow cytometry. Cryopreserved PBMC from R880F and R463F were thawed and rested in complete R10 medium (RPMI 1640 supplemented with 10% fetal bovine serum and penicillin/streptomycin) with benzoase for 2 h at 37°C, 5% CO_2_. Rested PBMC were then incubated in R10 medium containing 10 µg/ml GolgiPlug, 0.7 µg/ml GolgiStop, 1 µg/ml anti-human CD28 antibody and 1 µg/ml of anti-human CD49d antibody (BD Bioscience) at 1–2×10^6^/well in a 96-well V-bottomed plate. Cells were either left unstimulated, or stimulated with pools of overlapping peptides (2 µg/ml) corresponding to the autologous T/F virus Gag, N-terminal half of Env (Env1) or C-terminal half of Env (Env 2) sequence, or Staphylococcal Enterotoxin B (SEB, 1 µg/ml) as a positive control. The plate was incubated for 6 hours at 37°C, 5% CO_2_. After stimulation the cells were washed once with PBS and then stained with CD4-PE (Dako) for 10 min at room temperature (RT). The cells were then washed again with PBS, and then were stained with amine dye Aqua (Invitrogen) for 10 min at RT. The cells were then fixed with Cytofix/Cytoperm (BD Bioscience) for 15 min at RT, and washed once in PBS and once in permeabilisation buffer (BD Bioscience). They were then intracellularly stained with a CD154-FITC antibody (BD Bioscience) diluted in permeabilisation buffer for 30 min at RT. Finally, the cells were washed twice with permeabilisation buffer and then fixed with 4% paraformaldehyde solution. Samples were acquired on a CYAN flow cytometer (Dako) and were analysed using FlowJo (Treestar 8.7).

### Cloning of *env* genes from R880F and R463F and antibody neutralization assays

The SGA PCR amplification and cloning methods for obtaining HIV-1 *env* genes from virus present in plasma samples have been described elsewhere [94], [99], [102]. Briefly, full-length Env gp160 coding regions (plus Rev, Vpu, and partial Nef) were cloned into the CMV promoter-driven expression plasmid pcDNA3.1/V5-His-TOPO (Invitrogen) and a biological function screen for each clone was performed through generation of pseudoviruses following co-transfection with an Env-deficient subtype B proviral plasmid (pSG3Δenv) in 293T cells using FuGENE HD (Promega). The virus titers were defined by infecting TZM-bl cells, and the neutralization assay was performed as described previously [50]. In short, serial diluted heat-inactivated R880F/R463F plasma samples were assayed for neutralization potential against the Env pseudotyped viruses in the TZM-bl cell line, with luciferase activity as the ultimate readout, as described previously [102].

## Supporting Information

S1 Fig **Phylogenetic analysis of near full-length sequences of R463 and R880 transmission pairs in a neighbor-joining tree.** Sequences from the donor are shown in green; recipient sequences are shown in blue; reference sequences are shown in black. The R463F sequences were determined at day 12 post-Fiebig I/II, when the subject was at Fiebig stage IV, and the R880F sequences were determined at day 10 post-Fiebig I/II, when the subject was at Fiebig stage III. The sequences from both donors (R463M and R880M) were determined at the time-point when their linked transmission partners were at Fiebig stage IV and III respectively. The T/F virus population of both transmission pairs is derived from a single branch emanating from the donor sequences, confirming that a single variant from the donor population established infection. The scale represents the fractional pair-wise horizontal distance between sequences. Asterisks indicate bootstrap values of 90% or greater.
(PDF)

S2 Fig **qPCR competition replication assay for R880FVS and R463FVS.** Input virus concentrations were adjusted to a 1∶4 ratio of R463 and R880 RNA copies as assessed using qPCR quantitation described in Methods. Samples were removed on days 2, 4, 6, 8 and 10, and the relative percentage of each virus (genome equivalents) in the culture supernatants determined following qPCR quantitation.
(PDF)

S3 Fig **Highlighter plots of synonymous (green tick marks) and non-synonymous (red tick marks) changes over time from the consensus T/F virus near full-length sequence for (A) R880F and (B) R463F.** Sequence time points are indicated to the right, and are differentiated by shading. Gray bars indicate deletions in the amplified sequence. Where half-genome-length sequences were determined, the breakpoint between independent sequences is indicated by a slash.
(PDF)

S4 Fig **Investigation into viral escape in T cell epitopes recognized by individuals R880F (A) and R463F (B).** Serial dilutions of the indicated index sequence peptides (solid lines) and variants thereof containing amino acid changes selected for in the in vivo patient quasispecies (dotted lines) were tested for recognition by recipient PBMC in IFNγ ELISpot assays. The y-axis of each graph shows the magnitude of the response (spot-forming cells/10^6^ PBMC) detected to the peptide concentrations indicated on the x-axis (µM).
(PDF)

S5 Fig **Escape from early immunodominant epitopes in individuals R880F and R463F.** At the indicated time-points (days post-Fiebig I/II), the % of the primary HIV-specific T cell response that had been escaped was calculated by determining the % of the viral quasispecies that had undergone escape from the response to each epitope recognized by the primary T cell response (data in S1, S2 Tables, summarized in Table 3) and multiplying by the relative magnitude of the response concerned within the subject's primary HIV-specific T cell response (Table 3), then summing these values.
(PDF)

S6 Fig **Gag and Env-specific CD4+ T cell responses in subjects R880F and R463F.** Responses were analyzed at timepoints in acute and early infection, assessed by analysis of CD154 up-regulation in response to stimulation with autologous virus sequence-based peptide pools. (A) Dotplots illustrating the gating strategy for identification of antigen-responsive CD4+ T cells. Data from PBMCs cryopreserved from subject R880F at D73 post-Fiebig stage I/II stimulated with medium only or Env peptide pool 2 is shown. (B) Magnitude of the CD4+ T cell response (% CD4+ T cells up-regulating CD154) to Gag and Env peptide pools in subjects R880F and R463F at the indicated timepoints (days post-Fiebig stage I/II).
(PDF)

S7 Fig **Amino acid alignments for variable regions in Env gp120 over the first year of infection for (A) R880F and (B) R463F.** Sequences were compared to the T/F consensus sequence for each SC at the top of the alignments. Dots indicated that the residue was conserved, while dashes indicate a deletion/insertion. Amino acid substitutions from consensus are indicated. Gray stripes depict sites where mutations resulted in loss of a putative N-linked glycosylation motif (NxS/T); yellow stripes indicate the addition of putative N-linked glycosylation sites; cyan highlights in the consensus sequence identify stable N-linked glycosylation sites.
(PDF)

S1 Table **Longitudinal sequence analysis of epitopes in virus isolated from individual R880F.**
(PDF)

S2 Table **Longitudinal sequence analysis of epitopes in virus isolated from individual R463F.**
(PDF)

## References

[1] Buchbinder SP, Katz MH, Hessol NA, O'Malley PM, Holmberg SD (1994) Long-term HIV-1 infection without immunologic progression. AIDS 8: 1123–1128. 7986410 10.1097/00002030-199408000-00014

[2] Lobritz MA, Lassen KG, Arts EJ (2011) HIV-1 replicative fitness in elite controllers. Current opinion in HIV and AIDS 6: 214–220. 21430530 10.1097/COH.0b013e3283454cf5 PMC5730053

[3] Mellors JW, Kingsley LA, Rinaldo CR Jr, Todd JA, Hoo BS, et al (1995) Quantitation of HIV-1 RNA in plasma predicts outcome after seroconversion. Annals of internal medicine 122: 573–579. 7887550 10.7326/0003-4819-122-8-199504150-00003

[4] Cao Y, Qin L, Zhang L, Safrit J, Ho DD (1995) Virologic and immunologic characterization of long-term survivors of human immunodeficiency virus type 1 infection. The New England journal of medicine 332: 201–208. 7808485 10.1056/NEJM199501263320401

[5] Deeks SG, Walker BD (2007) Human immunodeficiency virus controllers: mechanisms of durable virus control in the absence of antiretroviral therapy. Immunity 27: 406–416. 17892849 10.1016/j.immuni.2007.08.010

[6] Munoz A, Kirby AJ, He YD, Margolick JB, Visscher BR, et al (1995) Long-term survivors with HIV-1 infection: incubation period and longitudinal patterns of CD4+ lymphocytes. Journal of acquired immune deficiency syndromes and human retrovirology: official publication of the International Retrovirology Association 8: 496–505. 10.1097/00042560-199504120-00010 7697447

[7] Sheppard HW, Lang W, Ascher MS, Vittinghoff E, Winkelstein W (1993) The characterization of non-progressors: long-term HIV-1 infection with stable CD4+ T-cell levels. AIDS 7: 1159–1166. 8105806

[8] Baker BM, Block BL, Rothchild AC, Walker BD (2009) Elite control of HIV infection: implications for vaccine design. Expert opinion on biological therapy 9: 55–69. 19063693 10.1517/14712590802571928 PMC3731764

[9] Okulicz JF, Marconi VC, Landrum ML, Wegner S, Weintrob A, et al (2009) Clinical outcomes of elite controllers, viremic controllers, and long-term nonprogressors in the US Department of Defense HIV natural history study. The Journal of infectious diseases 200: 1714–1723. 19852669 10.1086/646609

[10] Lambotte O, Boufassa F, Madec Y, Nguyen A, Goujard C, et al (2005) HIV controllers: a homogeneous group of HIV-1-infected patients with spontaneous control of viral replication. Clinical infectious diseases: an official publication of the Infectious Diseases Society of America 41: 1053–1056. 16142675 10.1086/433188

[11] McLaren PJ, Ripke S, Pelak K, Weintrob AC, Patsopoulos NA, et al (2012) Fine-mapping classical HLA variation associated with durable host control of HIV-1 infection in African Americans. Human molecular genetics 21: 4334–4347. 22718199 10.1093/hmg/dds226 PMC3441117

[12] O'Connell KA, Bailey JR, Blankson JN (2009) Elucidating the elite: mechanisms of control in HIV-1 infection. Trends in pharmacological sciences 30: 631–637. 19837464 10.1016/j.tips.2009.09.005

[13] Streeck H, Nixon DF (2010) T cell immunity in acute HIV-1 infection. The Journal of infectious diseases 202 Suppl 2: S302–308. 20846037 10.1086/655652 PMC2954287

[14] Jurriaans S, Van Gemen B, Weverling GJ, Van Strijp D, Nara P, et al (1994) The natural history of HIV-1 infection: virus load and virus phenotype independent determinants of clinical course? Virology 204: 223–233. 7916514 10.1006/viro.1994.1526

[15] Chopera DR, Woodman Z, Mlisana K, Mlotshwa M, Martin DP, et al (2008) Transmission of HIV-1 CTL escape variants provides HLA-mismatched recipients with a survival advantage. PLoS pathogens 4: e1000033. 18369479 10.1371/journal.ppat.1000033 PMC2265427

[16] Crawford H, Lumm W, Leslie A, Schaefer M, Boeras D, et al (2009) Evolution of HLA-B*5703 HIV-1 escape mutations in HLA-B*5703-positive individuals and their transmission recipients. J Exp Med 206: 909–921. 19307327 10.1084/jem.20081984 PMC2715113

[17] Goepfert PA, Lumm W, Farmer P, Matthews P, Prendergast A, et al (2008) Transmission of HIV-1 Gag immune escape mutations is associated with reduced viral load in linked recipients. The Journal of experimental medicine 205: 1009–1017. 18426987 10.1084/jem.20072457 PMC2373834

[18] Prince JL, Claiborne DT, Carlson JM, Schaefer M, Yu T, et al (2012) Role of Transmitted Gag CTL Polymorphisms in Defining Replicative Capacity and Early HIV-1 Pathogenesis. PLoS pathogens 8: e1003041. 23209412 10.1371/journal.ppat.1003041 PMC3510241

[19] Tang J, Malhotra R, Song W, Brill I, Hu L, et al (2010) Human leukocyte antigens and HIV type 1 viral load in early and chronic infection: predominance of evolving relationships. PloS one 5: e9629. 20224785 10.1371/journal.pone.0009629 PMC2835758

[20] Wright JK, Naidoo VL, Brumme ZL, Prince JL, Claiborne DT, et al (2012) Impact of HLA-B*81-associated mutations in HIV-1 Gag on viral replication capacity. Journal of virology 86: 3193–3199. 22238317 10.1128/JVI.06682-11 PMC3302318

[21] Yue L, Prentice HA, Farmer P, Song W, He D, et al (2013) Cumulative Impact of Host and Viral Factors on HIV-1 Viral-Load Control during Early Infection. Journal of virology 87: 708–715. 23115285 10.1128/JVI.02118-12 PMC3554094

[22] Pereyra F, Jia X, McLaren PJ, Telenti A, de Bakker PI, et al (2010) The major genetic determinants of HIV-1 control affect HLA class I peptide presentation. Science 330: 1551–1557. 21051598 10.1126/science.1195271 PMC3235490

[23] Fellay J, Shianna KV, Ge D, Colombo S, Ledergerber B, et al (2007) A whole-genome association study of major determinants for host control of HIV-1. Science 317: 944–947. 17641165 10.1126/science.1143767 PMC1991296

[24] Fellay J, Ge D, Shianna KV, Colombo S, Ledergerber B, et al (2009) Common genetic variation and the control of HIV-1 in humans. PLoS Genet 5: e1000791. 20041166 10.1371/journal.pgen.1000791 PMC2791220

[25] Tang J, Tang S, Lobashevsky E, Zulu I, Aldrovandi G, et al (2004) HLA allele sharing and HIV type 1 viremia in seroconverting Zambians with known transmitting partners. AIDS Research and Human Retroviruses 20: 19–25. 15000695 10.1089/088922204322749468

[26] Goulder PJ, Watkins DI (2008) Impact of MHC class I diversity on immune control of immunodeficiency virus replication. Nature reviews Immunology 8: 619–630. 10.1038/nri2357 PMC2963026 18617886

[27] Leslie A, Matthews PC, Listgarten J, Carlson JM, Kadie C, et al (2010) Additive contribution of HLA class I alleles in the immune control of HIV-1 infection. Journal of virology 84: 9879–9888. 20660184 10.1128/JVI.00320-10 PMC2937780

[28] Matthews PC, Adland E, Listgarten J, Leslie A, Mkhwanazi N, et al (2011) HLA-A*7401-mediated control of HIV viremia is independent of its linkage disequilibrium with HLA-B*5703. Journal of immunology 186: 5675–5686. 10.4049/jimmunol.1003711 PMC3738002 21498667

[29] Kiepiela P, Ngumbela K, Thobakgale C, Ramduth D, Honeyborne I, et al (2007) CD8+ T-cell responses to different HIV proteins have discordant associations with viral load. Nature medicine 13: 46–53. 10.1038/nm1520 17173051

[30] Shacklett BL (2010) Immune responses to HIV and SIV in mucosal tissues: ‘location, location, location’. Current opinion in HIV and AIDS 5: 128–134. 20543589 10.1097/COH.0b013e328335c178 PMC2886278

[31] Goulder PJ, Phillips RE, Colbert RA, McAdam S, Ogg G, et al (1997) Late escape from an immunodominant cytotoxic T-lymphocyte response associated with progression to AIDS. Nature medicine 3: 212–217. 10.1038/nm0297-212 9018241

[32] Leslie AJ, Pfafferott KJ, Chetty P, Draenert R, Addo MM, et al (2004) HIV evolution: CTL escape mutation and reversion after transmission. Nat Med 10: 282–289. 14770175 10.1038/nm992

[33] Goulder PJ, Watkins DI (2004) HIV and SIV CTL escape: implications for vaccine design. Nature reviews Immunology 4: 630–640. 10.1038/nri1417 15286729

[34] Borrow P, Lewicki H, Wei X, Horwitz MS, Peffer N, et al (1997) Antiviral pressure exerted by HIV-1-specific cytotoxic T lymphocytes (CTLs) during primary infection demonstrated by rapid selection of CTL escape virus. Nature medicine 3: 205–211. 10.1038/nm0297-205 9018240

[35] Jones NA, Wei X, Flower DR, Wong M, Michor F, et al (2004) Determinants of human immunodeficiency virus type 1 escape from the primary CD8+ cytotoxic T lymphocyte response. The Journal of experimental medicine 200: 1243–1256. 15545352 10.1084/jem.20040511 PMC2211924

[36] Brockman MA, Brumme ZL, Brumme CJ, Miura T, Sela J, et al (2010) Early selection in Gag by protective HLA alleles contributes to reduced HIV-1 replication capacity that may be largely compensated for in chronic infection. Journal of virology 84: 11937–11949. 20810731 10.1128/JVI.01086-10 PMC2977869

[37] Miura T, Brockman MA, Schneidewind A, Lobritz M, Pereyra F, et al (2009) HLA-B57/B*5801 human immunodeficiency virus type 1 elite controllers select for rare gag variants associated with reduced viral replication capacity and strong cytotoxic T-lymphocyte [corrected] recognition. Journal of virology 83: 2743–2755. 19116253 10.1128/JVI.02265-08 PMC2648254

[38] Bailey JR, Williams TM, Siliciano RF, Blankson JN (2006) Maintenance of viral suppression in HIV-1-infected HLA-B*57+ elite suppressors despite CTL escape mutations. The Journal of experimental medicine 203: 1357–1369. 16682496 10.1084/jem.20052319 PMC2121215

[39] Alizon S, von Wyl V, Stadler T, Kouyos RD, Yerly S, et al (2010) Phylogenetic approach reveals that virus genotype largely determines HIV set-point viral load. PLoS pathogens 6: e1001123. 20941398 10.1371/journal.ppat.1001123 PMC2947993

[40] Hecht FM, Hartogensis W, Bragg L, Bacchetti P, Atchison R, et al (2010) HIV RNA level in early infection is predicted by viral load in the transmission source. AIDS 24: 941–945. 20168202 10.1097/QAD.0b013e328337b12e PMC2887742

[41] Hollingsworth TD, Laeyendecker O, Shirreff G, Donnelly CA, Serwadda D, et al (2010) HIV-1 transmitting couples have similar viral load set-points in Rakai, Uganda. PLoS Pathog 6: e1000876. 20463808 10.1371/journal.ppat.1000876 PMC2865511

[42] Lyles RH, Munoz A, Yamashita TE, Bazmi H, Detels R, et al (2000) Natural history of human immunodeficiency virus type 1 viremia after seroconversion and proximal to AIDS in a large cohort of homosexual men. Multicenter AIDS Cohort Study. The Journal of infectious diseases 181: 872–880. 10720507 10.1086/315339

[43] Wright JK, Novitsky V, Brockman MA, Brumme ZL, Brumme CJ, et al (2011) Influence of Gag-protease-mediated replication capacity on disease progression in individuals recently infected with HIV-1 subtype C. Journal of virology 85: 3996–4006. 21289112 10.1128/JVI.02520-10 PMC3126116

[44] Miura T, Brockman MA, Brumme CJ, Brumme ZL, Carlson JM, et al (2008) Genetic characterization of human immunodeficiency virus type 1 in elite controllers: lack of gross genetic defects or common amino acid changes. Journal of virology 82: 8422–8430. 18562530 10.1128/JVI.00535-08 PMC2519665

[45] Miura T, Brockman MA, Brumme ZL, Brumme CJ, Pereyra F, et al (2009) HLA-associated alterations in replication capacity of chimeric NL4-3 viruses carrying gag-protease from elite controllers of human immunodeficiency virus type 1. Journal of virology 83: 140–149. 18971283 10.1128/JVI.01471-08 PMC2612337

[46] Miura T, Brumme ZL, Brockman MA, Rosato P, Sela J, et al (2010) Impaired replication capacity of acute/early viruses in persons who become HIV controllers. Journal of virology 84: 7581–7591. 20504921 10.1128/JVI.00286-10 PMC2897600

[47] Lassen KG, Lobritz MA, Bailey JR, Johnston S, Nguyen S, et al (2009) Elite suppressor-derived HIV-1 envelope glycoproteins exhibit reduced entry efficiency and kinetics. PLoS pathogens 5: e1000377. 19360131 10.1371/journal.ppat.1000377 PMC2661022

[48] Blankson JN, Bailey JR, Thayil S, Yang HC, Lassen K, et al (2007) Isolation and characterization of replication-competent human immunodeficiency virus type 1 from a subset of elite suppressors. Journal of virology 81: 2508–2518. 17151109 10.1128/JVI.02165-06 PMC1865922

[49] Boeras DI, Hraber PT, Hurlston M, Evans-Strickfaden T, Bhattacharya T, et al (2011) Role of donor genital tract HIV-1 diversity in the transmission bottleneck. Proceedings of the National Academy of Sciences of the United States of America 108: E1156–1163. 22065783 10.1073/pnas.1103764108 PMC3219102

[50] Derdeyn CA, Decker JM, Bibollet-Ruche F, Mokili JL, Muldoon M, et al (2004) Envelope-constrained neutralization-sensitive HIV-1 after heterosexual transmission. Science 303: 2019–2022. 15044802 10.1126/science.1093137

[51] Haaland RE, Hawkins PA, Salazar-Gonzalez J, Johnson A, Tichacek A, et al (2009) Inflammatory genital infections mitigate a severe genetic bottleneck in heterosexual transmission of subtype A and C HIV-1. PLoS pathogens 5: e1000274. 19165325 10.1371/journal.ppat.1000274 PMC2621345

[52] Rousseau CM, Birditt BA, McKay AR, Stoddard JN, Lee TC, et al (2006) Large-scale amplification, cloning and sequencing of near full-length HIV-1 subtype C genomes. Journal of virological methods 136: 118–125. 16701907 10.1016/j.jviromet.2006.04.009

[53] Salazar-Gonzalez JF, Salazar MG, Keele BF, Learn GH, Giorgi EE, et al (2009) Genetic identity, biological phenotype, and evolutionary pathways of transmitted/founder viruses in acute and early HIV-1 infection. The Journal of experimental medicine 206: 1273–1289. 19487424 10.1084/jem.20090378 PMC2715054

[54] Goonetilleke N, Liu MK, Salazar-Gonzalez JF, Ferrari G, Giorgi E, et al (2009) The first T cell response to transmitted/founder virus contributes to the control of acute viremia in HIV-1 infection. The Journal of experimental medicine 206: 1253–1272. 19487423 10.1084/jem.20090365 PMC2715063

[55] Bar KJ, Tsao CY, Iyer SS, Decker JM, Yang Y, et al (2012) Early low-titer neutralizing antibodies impede HIV-1 replication and select for virus escape. PLoS pathogens 8: e1002721. 22693447 10.1371/journal.ppat.1002721 PMC3364956

[56] Baalwa J, Wang S, Parrish NF, Decker JM, Keele BF, et al (2013) Molecular identification, cloning and characterization of transmitted/founder HIV-1 subtype A, D and A/D infectious molecular clones. Virology 436: 33–48. 23123038 10.1016/j.virol.2012.10.009 PMC3545109

[57] Boutwell CL, Carlson JM, Lin TH, Seese A, Power KA, et al (2013) Frequent and Variable Cytotoxic-T-Lymphocyte Escape-associated Fitness Costs in the Human Immunodeficiency Virus Type 1 Subtype B Gag Proteins. Journal of virology 10.1128/JVI.03233-12 PMC3624202 23365420

[58] Brockman MA, Schneidewind A, Lahaie M, Schmidt A, Miura T, et al (2007) Escape and compensation from early HLA-B57-mediated cytotoxic T-lymphocyte pressure on human immunodeficiency virus type 1 Gag alter capsid interactions with cyclophilin A. Journal of virology 81: 12608–12618. 17728232 10.1128/JVI.01369-07 PMC2169025

[59] Crawford H, Prado JG, Leslie A, Hue S, Honeyborne I, et al (2007) Compensatory mutation partially restores fitness and delays reversion of escape mutation within the immunodominant HLA-B*5703-restricted Gag epitope in chronic human immunodeficiency virus type 1 infection. Journal of virology 81: 8346–8351. 17507468 10.1128/JVI.00465-07 PMC1951305

[60] Jin X, Bauer DE, Tuttleton SE, Lewin S, Gettie A, et al (1999) Dramatic rise in plasma viremia after CD8(+) T cell depletion in simian immunodeficiency virus-infected macaques. The Journal of experimental medicine 189: 991–998. 10075982 10.1084/jem.189.6.991 PMC2193038

[61] Schmitz JE, Kuroda MJ, Santra S, Sasseville VG, Simon MA, et al (1999) Control of viremia in simian immunodeficiency virus infection by CD8+ lymphocytes. Science 283: 857–860. 9933172 10.1126/science.283.5403.857

[62] Liu MK, Hawkins N, Ritchie AJ, Ganusov VV, Whale V, et al (2013) Vertical T cell immunodominance and epitope entropy determine HIV-1 escape. J Clin Invest 123: 380–393. 23221345 10.1172/JCI65330 PMC3533301

[63] Mothe B, Llano A, Ibarrondo J, Daniels M, Miranda C, et al (2011) Definition of the viral targets of protective HIV-1-specific T cell responses. J Transl Med 9: 208. 22152067 10.1186/1479-5876-9-208 PMC3292987

[64] Payne RP, Kloverpris H, Sacha JB, Brumme Z, Brumme C, et al (2010) Efficacious early antiviral activity of HIV Gag- and Pol-specific HLA-B 2705-restricted CD8+ T cells. Journal of virology 84: 10543–10557. 20686036 10.1128/JVI.00793-10 PMC2950555

[65] Ferrari G, Korber B, Goonetilleke N, Liu MK, Turnbull EL, et al (2011) Relationship between functional profile of HIV-1 specific CD8 T cells and epitope variability with the selection of escape mutants in acute HIV-1 infection. PLoS pathogens 7: e1001273. 21347345 10.1371/journal.ppat.1001273 PMC3037354

[66] Derdeyn CA, Decker JM, Sfakianos JN, Wu X, O'Brien WA, et al (2000) Sensitivity of human immunodeficiency virus type 1 to the fusion inhibitor T-20 is modulated by coreceptor specificity defined by the V3 loop of gp120. J Virol 74: 8358–8367. 10954535 10.1128/jvi.74.18.8358-8367.2000 PMC116346

[67] Wei X, Decker JM, Liu H, Zhang Z, Arani RB, et al (2002) Emergence of resistant human immunodeficiency virus type 1 in patients receiving fusion inhibitor (T-20) monotherapy. Antimicrob Agents Chemother 46: 1896–1905. 12019106 10.1128/AAC.46.6.1896-1905.2002 PMC127242

[68] Borrow P, Lewicki H, Hahn BH, Shaw GM, Oldstone MB (1994) Virus-specific CD8+ cytotoxic T-lymphocyte activity associated with control of viremia in primary human immunodeficiency virus type 1 infection. Journal of virology 68: 6103–6110. 8057491 10.1128/jvi.68.9.6103-6110.1994 PMC237022

[69] Koup RA, Safrit JT, Cao Y, Andrews CA, McLeod G, et al (1994) Temporal association of cellular immune responses with the initial control of viremia in primary human immunodeficiency virus type 1 syndrome. Journal of virology 68: 4650–4655. 8207839 10.1128/jvi.68.7.4650-4655.1994 PMC236393

[70] Turnbull EL, Wong M, Wang S, Wei X, Jones NA, et al (2009) Kinetics of expansion of epitope-specific T cell responses during primary HIV-1 infection. Journal of immunology 182: 7131–7145. 10.4049/jimmunol.0803658 19454710

[71] Fiebig EW, Wright DJ, Rawal BD, Garrett PE, Schumacher RT, et al (2003) Dynamics of HIV viremia and antibody seroconversion in plasma donors: implications for diagnosis and staging of primary HIV infection. AIDS 17: 1871–1879. 12960819 10.1097/00002030-200309050-00005

[72] Hatano H, Delwart EL, Norris PJ, Lee TH, Dunn-Williams J, et al (2009) Evidence for persistent low-level viremia in individuals who control human immunodeficiency virus in the absence of antiretroviral therapy. Journal of virology 83: 329–335. 18945778 10.1128/JVI.01763-08 PMC2612329

[73] Navis M, Schellens I, van Baarle D, Borghans J, van Swieten P, et al (2007) Viral replication capacity as a correlate of HLA B57/B5801-associated nonprogressive HIV-1 infection. Journal of immunology 179: 3133–3143. 10.4049/jimmunol.179.5.3133 17709528

[74] Janssen EM, Lemmens EE, Wolfe T, Christen U, von Herrath MG, et al (2003) CD4+ T cells are required for secondary expansion and memory in CD8+ T lymphocytes. Nature 421: 852–856. 12594515 10.1038/nature01441

[75] Betts MR, Nason MC, West SM, De Rosa SC, Migueles SA, et al (2006) HIV nonprogressors preferentially maintain highly functional HIV-specific CD8+ T cells. Blood 107: 4781–4789. 16467198 10.1182/blood-2005-12-4818 PMC1895811

[76] Migueles SA, Osborne CM, Royce C, Compton AA, Joshi RP, et al (2008) Lytic granule loading of CD8+ T cells is required for HIV-infected cell elimination associated with immune control. Immunity 29: 1009–1021. 19062316 10.1016/j.immuni.2008.10.010 PMC2622434

[77] Ribeiro-dos-Santos P, Turnbull EL, Monteiro M, Legrand A, Conrod K, et al (2012) Chronic HIV infection affects the expression of the 2 transcription factors required for CD8 T-cell differentiation into cytolytic effectors. Blood 119: 4928–4938. 22490682 10.1182/blood-2011-12-395186

[78] Apps R, Qi Y, Carlson JM, Chen HY, Gao XJ, et al (2013) Influence of HLA-C Expression Level on HIV Control. Science 340: 87–91. 23559252 10.1126/science.1232685 PMC3784322

[79] Addo MM, Yu XG, Rathod A, Cohen D, Eldridge RL, et al (2003) Comprehensive epitope analysis of human immunodeficiency virus type 1 (HIV-1)-specific T-cell responses directed against the entire expressed HIV-1 genome demonstrate broadly directed responses, but no correlation to viral load. Journal of virology 77: 2081–2092. 12525643 10.1128/JVI.77.3.2081-2092.2003 PMC140965

[80] Ganusov VV, Goonetilleke N, Liu MK, Ferrari G, Shaw GM, et al (2011) Fitness costs and diversity of the cytotoxic T lymphocyte (CTL) response determine the rate of CTL escape during acute and chronic phases of HIV infection. Journal of virology 85: 10518–10528. 21835793 10.1128/JVI.00655-11 PMC3187476

[81] van Deutekom HW, Wijnker G, de Boer RJ (2013) The rate of immune escape vanishes when multiple immune responses control an HIV infection. Journal of immunology 191: 3277–3286. 10.4049/jimmunol.1300962 23940274

[82] Mudd PA, Ericsen AJ, Burwitz BJ, Wilson NA, O'Connor DH, et al (2012) Escape from CD8(+) T cell responses in Mamu-B*00801(+) macaques differentiates progressors from elite controllers. Journal of immunology 188: 3364–3370. 10.4049/jimmunol.1102470 PMC3883130 22387557

[83] Wang YE, Li B, Carlson JM, Streeck H, Gladden AD, et al (2009) Protective HLA class I alleles that restrict acute-phase CD8+ T-cell responses are associated with viral escape mutations located in highly conserved regions of human immunodeficiency virus type 1. Journal of virology 83: 1845–1855. 19036810 10.1128/JVI.01061-08 PMC2643763

[84] Gasper-Smith N, Crossman DM, Whitesides JF, Mensali N, Ottinger JS, et al (2008) Induction of plasma (TRAIL), TNFR-2, Fas ligand, and plasma microparticles after human immunodeficiency virus type 1 (HIV-1) transmission: implications for HIV-1 vaccine design. Journal of virology 82: 7700–7710. 18508902 10.1128/JVI.00605-08 PMC2493338

[85] Frleta D, Ochoa CE, Kramer HB, Khan SA, Stacey AR, et al (2012) HIV-1 infection-induced apoptotic microparticles inhibit human DCs via CD44. J Clin Invest 122: 4685–4697. 23160198 10.1172/JCI64439 PMC3533550

[86] Overbaugh J, Morris L (2012) The Antibody Response against HIV-1. Cold Spring Harbor perspectives in medicine 2: a007039. 22315717 10.1101/cshperspect.a007039 PMC3253031

[87] Montefiori DC, Pantaleo G, Fink LM, Zhou JT, Zhou JY, et al (1996) Neutralizing and infection-enhancing antibody responses to human immunodeficiency virus type 1 in long-term nonprogressors. The Journal of infectious diseases 173: 60–67. 8537683 10.1093/infdis/173.1.60

[88] Carotenuto P, Looij D, Keldermans L, de Wolf F, Goudsmit J (1998) Neutralizing antibodies are positively associated with CD4+ T-cell counts and T-cell function in long-term AIDS-free infection. AIDS 12: 1591–1600. 9764777 10.1097/00002030-199813000-00005

[89] Cecilia D, Kleeberger C, Munoz A, Giorgi JV, Zolla-Pazner S (1999) A longitudinal study of neutralizing antibodies and disease progression in HIV-1-infected subjects. The Journal of infectious diseases 179: 1365–1374. 10228056 10.1086/314773

[90] Pereyra F, Addo MM, Kaufmann DE, Liu Y, Miura T, et al (2008) Genetic and immunologic heterogeneity among persons who control HIV infection in the absence of therapy. The Journal of infectious diseases 197: 563–571. 18275276 10.1086/526786

[91] Doria-Rose NA, Klein RM, Manion MM, O'Dell S, Phogat A, et al (2009) Frequency and phenotype of human immunodeficiency virus envelope-specific B cells from patients with broadly cross-neutralizing antibodies. Journal of virology 83: 188–199. 18922865 10.1128/JVI.01583-08 PMC2612342

[92] Lambotte O, Ferrari G, Moog C, Yates NL, Liao HX, et al (2009) Heterogeneous neutralizing antibody and antibody-dependent cell cytotoxicity responses in HIV-1 elite controllers. AIDS 23: 897–906. 19414990 10.1097/QAD.0b013e328329f97d PMC3652655

[93] Mahalanabis M, Jayaraman P, Miura T, Pereyra F, Chester EM, et al (2009) Continuous viral escape and selection by autologous neutralizing antibodies in drug-naive human immunodeficiency virus controllers. Journal of virology 83: 662–672. 18987151 10.1128/JVI.01328-08 PMC2612349

[94] Murphy MK, Yue L, Pan R, Boliar S, Sethi A, et al (2013) Viral Escape from Neutralizing Antibodies in Early Subtype A HIV-1 Infection Drives an Increase in Autologous Neutralization Breadth. PLoS Pathog 9: e1003173. 23468623 10.1371/journal.ppat.1003173 PMC3585129

[95] Overbaugh J, Rudensey LM (1992) Alterations in potential sites for glycosylation predominate during evolution of the simian immunodeficiency virus envelope gene in macaques. Journal of virology 66: 5937–5948. 1527847 10.1128/jvi.66.10.5937-5948.1992 PMC241471

[96] Chackerian B, Rudensey LM, Overbaugh J (1997) Specific N-linked and O-linked glycosylation modifications in the envelope V1 domain of simian immunodeficiency virus variants that evolve in the host alter recognition by neutralizing antibodies. Journal of virology 71: 7719–7727. 9311856 10.1128/jvi.71.10.7719-7727.1997 PMC192123

[97] Richman DD, Wrin T, Little SJ, Petropoulos CJ (2003) Rapid evolution of the neutralizing antibody response to HIV type 1 infection. Proceedings of the National Academy of Sciences of the United States of America 100: 4144–4149. 12644702 10.1073/pnas.0630530100 PMC153062

[98] Wei X, Decker JM, Wang S, Hui H, Kappes JC, et al (2003) Antibody neutralization and escape by HIV-1. Nature 422: 307–312. 12646921 10.1038/nature01470

[99] Li B, Decker JM, Johnson RW, Bibollet-Ruche F, Wei X, et al (2006) Evidence for potent autologous neutralizing antibody titers and compact envelopes in early infection with subtype C human immunodeficiency virus type 1. Journal of virology 80: 5211–5218. 16699001 10.1128/JVI.00201-06 PMC1472127

[100] Bunnik EM, Pisas L, van Nuenen AC, Schuitemaker H (2008) Autologous neutralizing humoral immunity and evolution of the viral envelope in the course of subtype B human immunodeficiency virus type 1 infection. Journal of virology 82: 7932–7941. 18524815 10.1128/JVI.00757-08 PMC2519599

[101] Moore PL, Gray ES, Morris L (2009) Specificity of the autologous neutralizing antibody response. Current opinion in HIV and AIDS 4: 358–363. 20048698 10.1097/COH.0b013e32832ea7e8 PMC3004050

[102] Rong R, Li B, Lynch RM, Haaland RE, Murphy MK, et al (2009) Escape from autologous neutralizing antibodies in acute/early subtype C HIV-1 infection requires multiple pathways. PLoS pathogens 5: e1000594. 19763269 10.1371/journal.ppat.1000594 PMC2741593

[103] Gnanakaran S, Lang D, Daniels M, Bhattacharya T, Derdeyn CA, et al (2007) Clade-specific differences between human immunodeficiency virus type 1 clades B and C: diversity and correlations in C3-V4 regions of gp120. Journal of virology 81: 4886–4891. 17166900 10.1128/JVI.01954-06 PMC1900169

[104] Lynch RM, Rong R, Boliar S, Sethi A, Li B, et al (2011) The B cell response is redundant and highly focused on V1V2 during early subtype C infection in a Zambian seroconverter. J Virol 85: 905–915. 20980495 10.1128/JVI.02006-10 PMC3020014

[105] Trask SA, Derdeyn CA, Fideli U, Chen Y, Meleth S, et al (2002) Molecular epidemiology of human immunodeficiency virus type 1 transmission in a heterosexual cohort of discordant couples in Zambia. Journal of virology 76: 397–405. 11739704 10.1128/JVI.76.1.397-405.2002 PMC135722

[106] Keele BF, Giorgi EE, Salazar-Gonzalez JF, Decker JM, Pham KT, et al (2008) Identification and characterization of transmitted and early founder virus envelopes in primary HIV-1 infection. Proceedings of the National Academy of Sciences of the United States of America 105: 7552–7557. 18490657 10.1073/pnas.0802203105 PMC2387184

[107] Roederer M, Koup RA (2003) Optimized determination of T cell epitope responses. Journal of immunological methods 274: 221–228. 12609547 10.1016/s0022-1759(02)00423-4

[108] Pantaleo G, Soudeyns H, Demarest JF, Vaccarezza M, Graziosi C, et al (1997) Evidence for rapid disappearance of initially expanded HIV-specific CD8+ T cell clones during primary HIV infection. Proceedings of the National Academy of Sciences of the United States of America 94: 9848–9853. 9275214 10.1073/pnas.94.18.9848 PMC23280

[109] Poss M, Gosink J, Thomas E, Kreiss JK, Ndinya-Achola J, et al (1997) Phylogenetic evaluation of Kenyan HIV type 1 isolates. AIDS Res Hum Retroviruses 13: 493–499. 9100991 10.1089/aid.1997.13.493

