# Supplementary figures and images for: Transmitted Virus Fitness and Host T Cell Responses Collectively Define Divergent Infection Outcomes in Two HIV-1 Recipients
Source: PLoS Pathog. 2015 Jan 8;11(1):e1004565. doi: 10.1371/journal.ppat.1004565 (PMC4287535; doi:10.1371/journal.ppat.1004565)

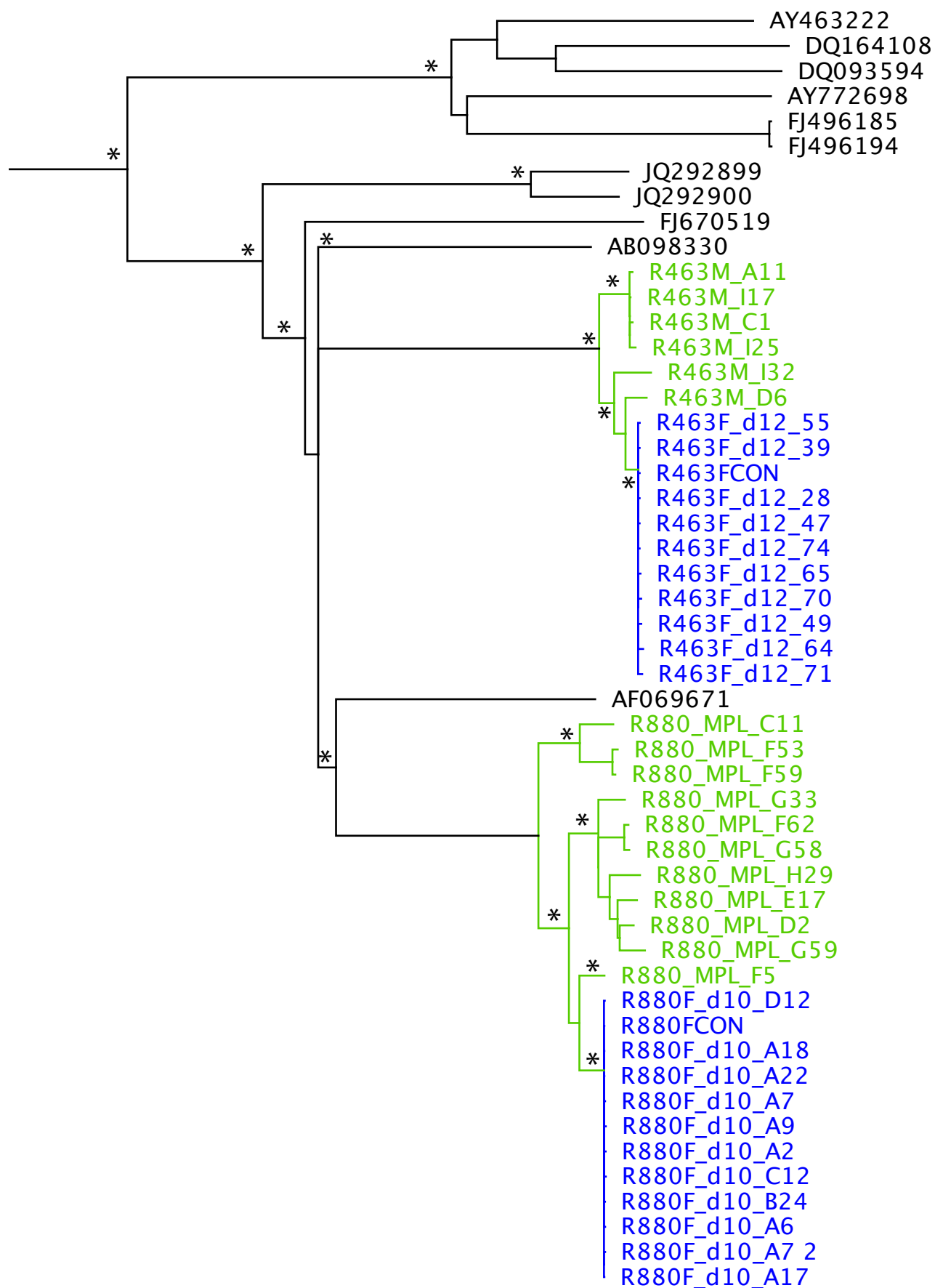

Supplement: S1 Fig — Phylogenetic analysis of near full-length sequences of R463 and R880 transmission pairs in a neighbor-joining tree. Sequences from the donor are shown in green; recipient sequences are shown in blue; reference sequences are shown in black. The R463F sequences were determined at day 12 post-Fiebig I/II, when the subject was at Fiebig stage IV, and the R880F sequences were determined at day 10 post-Fiebig I/II, when the subject was at Fiebig stage III. The sequences from both donors (R463M and R880M) were determined at the time-point when their linked transmission partners were at Fiebig stage IV and III respectively. The T/F virus population of both transmission pairs is derived from a single branch emanating from the donor sequences, confirming that a single variant from the donor population established infection. The scale represents the fractional pair-wise horizontal distance between sequences. Asterisks indicate bootstrap values of 90% or greater. (PDF) [file ppat.1004565.s001.pdf]

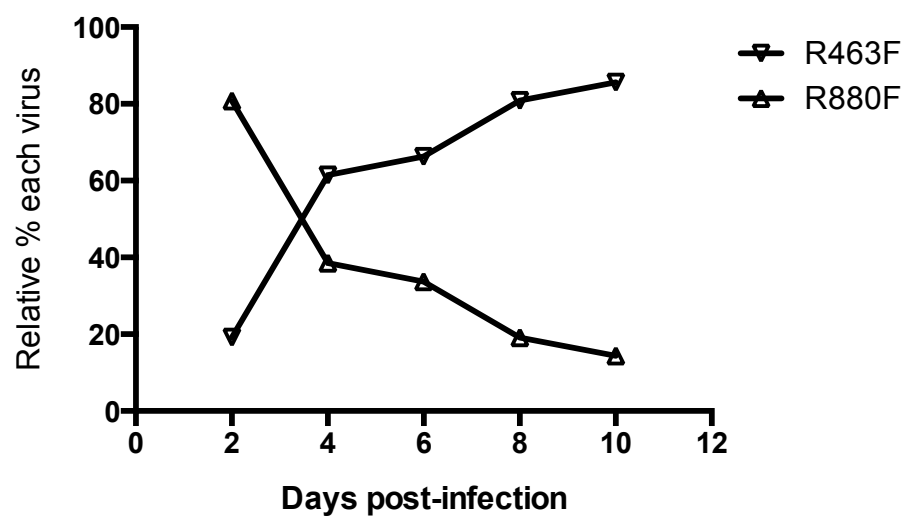

Supplement: S2 Fig — qPCR competition replication assay for R880FVS and R463FVS. Input virus concentrations were adjusted to a 1∶4 ratio of R463 and R880 RNA copies as assessed using qPCR quantitation described in Methods. Samples were removed on days 2, 4, 6, 8 and 10, and the relative percentage of each virus (genome equivalents) in the culture supernatants determined following qPCR quantitation. (PDF) [file ppat.1004565.s002.pdf]

A.

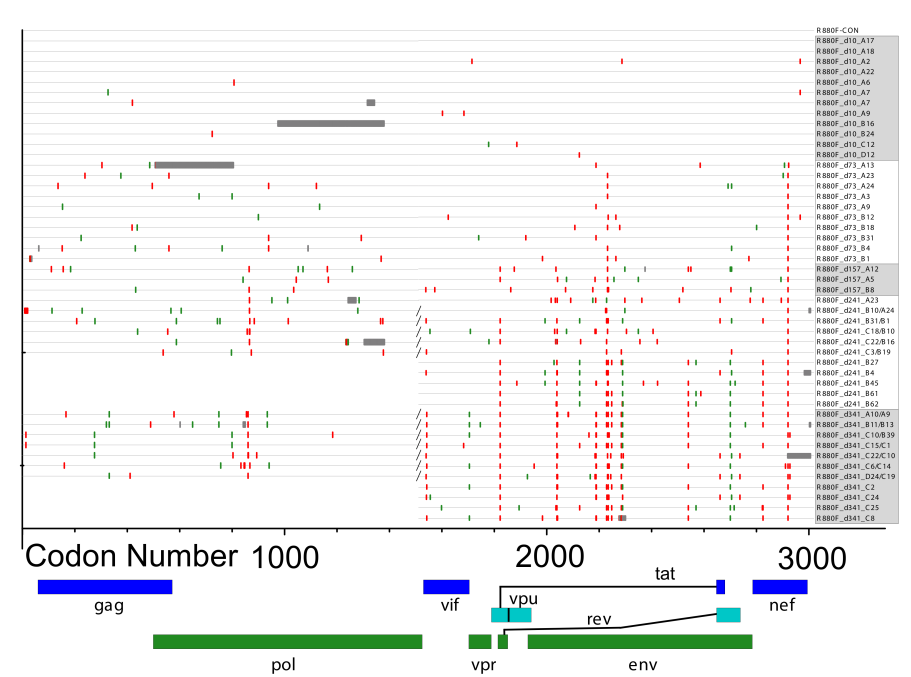

B.

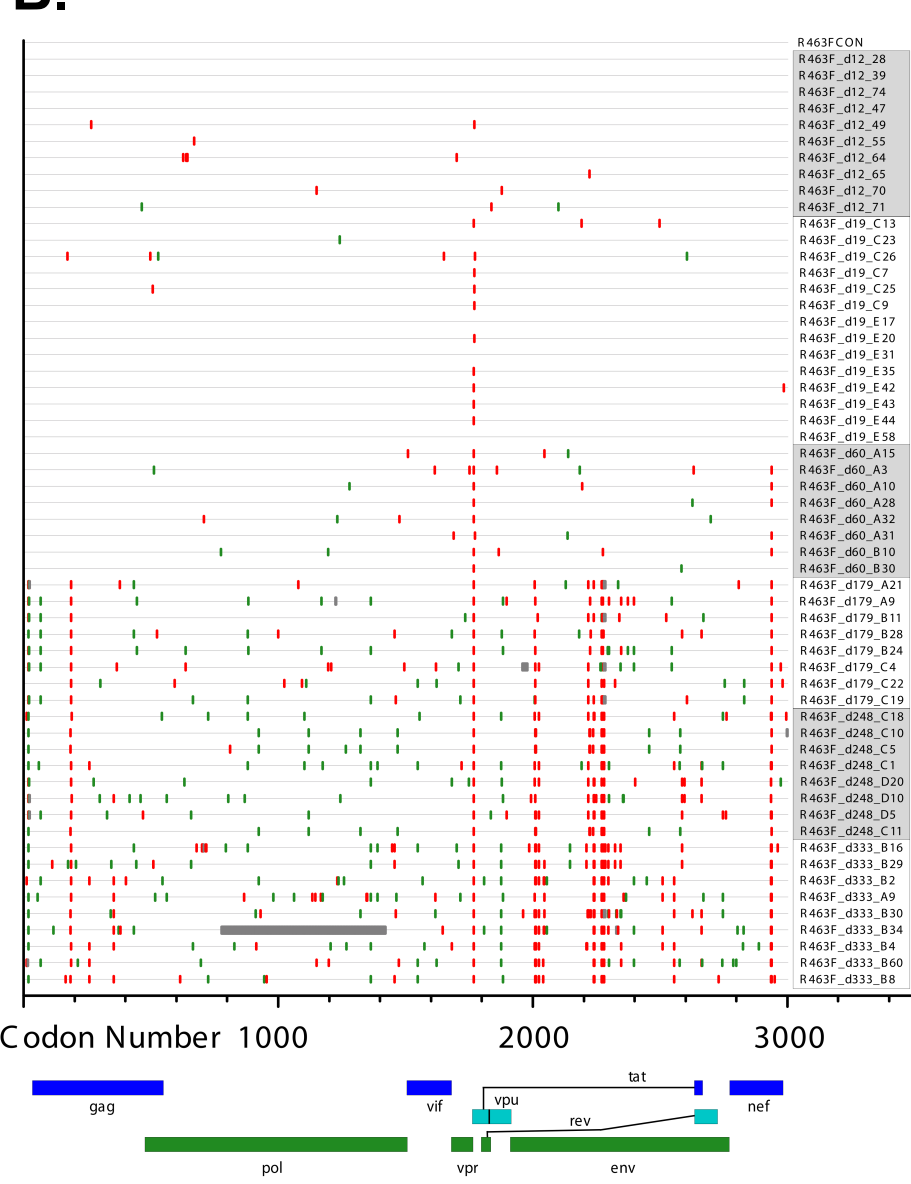

Supplement: S3 Fig — Highlighter plots of synonymous (green tick marks) and non-synonymous (red tick marks) changes over time from the consensus T/F virus near full-length sequence for (A) R880F and (B) R463F. Sequence time points are indicated to the right, and are differentiated by shading. Gray bars indicate deletions in the amplified sequence. Where half-genome-length sequences were determined, the breakpoint between independent sequences is indicated by a slash. (PDF) [file ppat.1004565.s003.pdf]

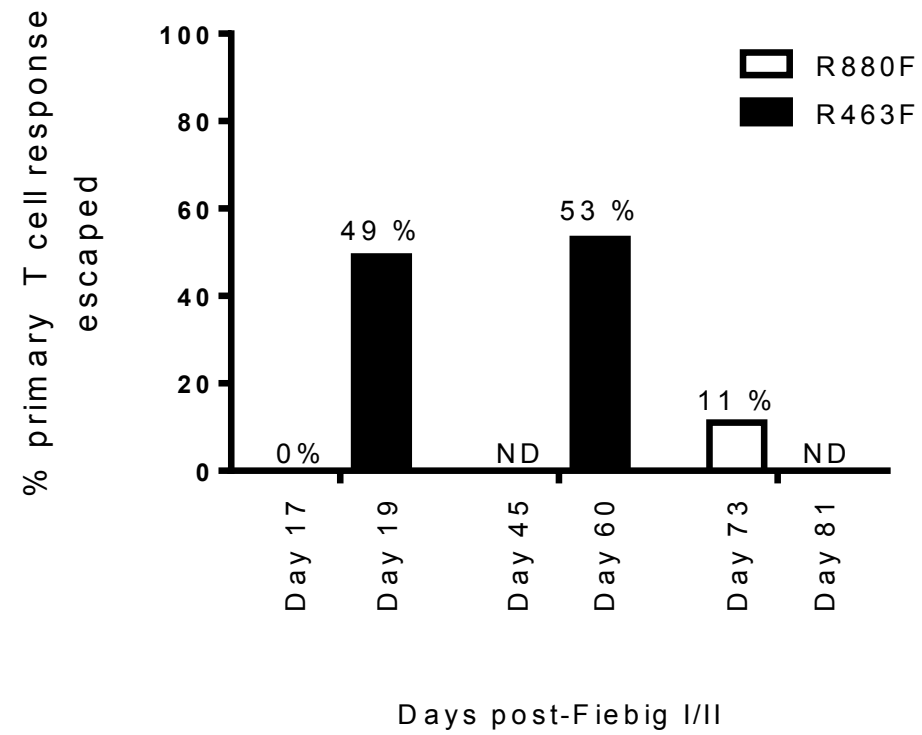

Supplement: S5 Fig — Escape from early immunodominant epitopes in individuals R880F and R463F. At the indicated time-points (days post-Fiebig I/II), the % of the primary HIV-specific T cell response that had been escaped was calculated by determining the % of the viral quasispecies that had undergone escape from the response to each epitope recognized by the primary T cell response (data in S1, S2 Tables, summarized in Table 3) and multiplying by the relative magnitude of the response concerned within the subject's primary HIV-specific T cell response (Table 3), then summing these values. (PDF) [file ppat.1004565.s005.pdf]

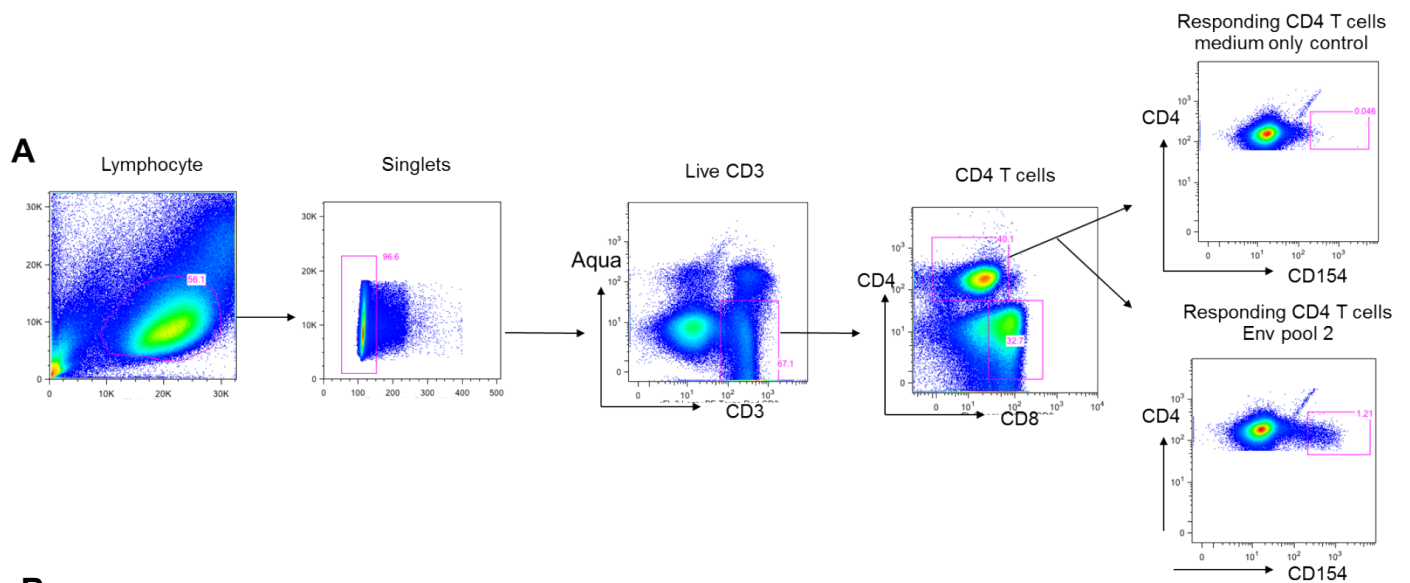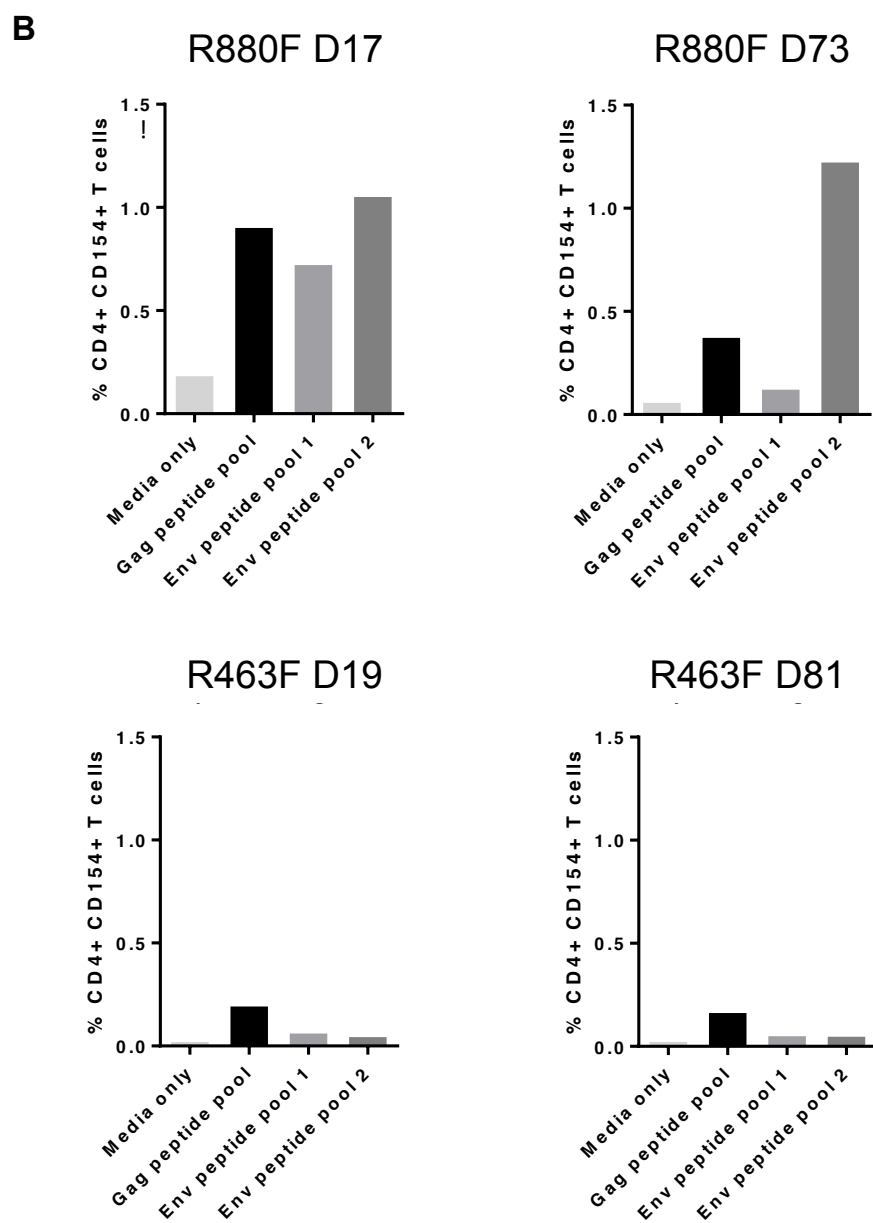

Supplement: S6 Fig — Gag and Env-specific CD4+ T cell responses in subjects R880F and R463F. Responses were analyzed at timepoints in acute and early infection, assessed by analysis of CD154 up-regulation in response to stimulation with autologous virus sequence-based peptide pools. (A) Dotplots illustrating the gating strategy for identification of antigen-responsive CD4+ T cells. Data from PBMCs cryopreserved from subject R880F at D73 post-Fiebig stage I/II stimulated with medium only or Env peptide pool 2 is shown. (B) Magnitude of the CD4+ T cell response (% CD4+ T cells up-regulating CD154) to Gag and Env peptide pools in subjects R880F and R463F at the indicated timepoints (days post-Fiebig stage I/II). (PDF) [file ppat.1004565.s006.pdf]
