# Supplementary material for: Transmitted Virus Fitness and Host T Cell Responses Collectively Define Divergent Infection Outcomes in Two HIV-1 Recipients
Source: PLoS Pathog. 2015 Jan 8;11(1):e1004565. doi: 10.1371/journal.ppat.1004565 (PMC4287535; doi:10.1371/journal.ppat.1004565)
Supplement: S4 Fig — Investigation into viral escape in T cell epitopes recognized by individuals R880F (A) and R463F (B). Serial dilutions of the indicated index sequence peptides (solid lines) and variants thereof containing amino acid changes selected for in the in vivo patient quasispecies (dotted lines) were tested for recognition by recipient PBMC in IFNγ ELISpot assays. The y-axis of each graph shows the magnitude of the response (spot-forming cells/106 PBMC) detected to the peptide concentrations indicated on the x-axis (µM). (PDF) [file ppat.1004565.s004.pdf]

A.

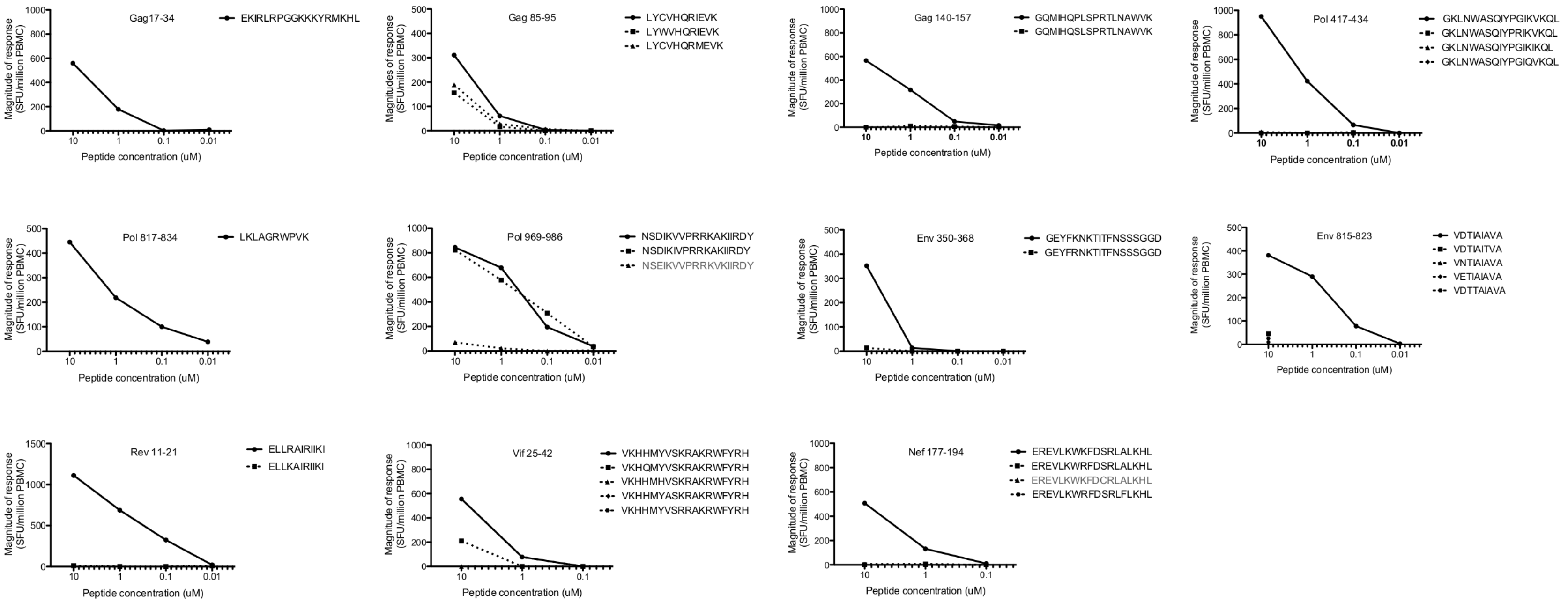
Magnitude of response (SFU/million PBMC)
Peptide concentration (uM)
Gag17-34
EKIRLRPGGKKKYRMKHL
Gag 85-95
LYCVHQRIEVK
LYWVHQRIEVK
LYCVHQRMEVK
Gag 140-157
GQMIHQPLSPRTLNAWVK
GQMIHQSLSPRTLNAWVK
Pol 417-434
GKLNWASQIYPGIKVKQL
GKLNWASQIYPRIKVKQL
GKLNWASQIYPGIKIKQL
GKLNWASQIYPGIQVKQL
Pol 817-834
LKLAGRWPVK
Pol 969-986
NSDIKVVPRRKAKIIRDY
NSDIKIVPRRKAKIIRDY
NSEIKVVPRRKVKIIRDY
Env 350-368
GEYFKNKTITFNSSSGGD
GEYFRNKTITFNSSSGGD
Env 815-823
VDTIAIAVA
VDTIAITVA
VNTIAIAVA
VETIAIAVA
VDTTAIAVA
Rev 11-21
ELLRAIRIIKI
ELLKAIRIIKI
Vif 25-42
VKHHMYVSKRAKRWFYRH
VKHQMYVSKRAKRWFYRH
VKHHMHVSKRAKRWFYRH
VKHHMYASKRAKRWFYRH
VKHHMYVSRRAKRWFYRH
Nef 177-194
EREVLKWKFDSRLALKHL
EREVLKWRFDSRLALKHL
EREVLKWKFDCRLALKHL
EREVLKWRFDSRLFLKHL

B.

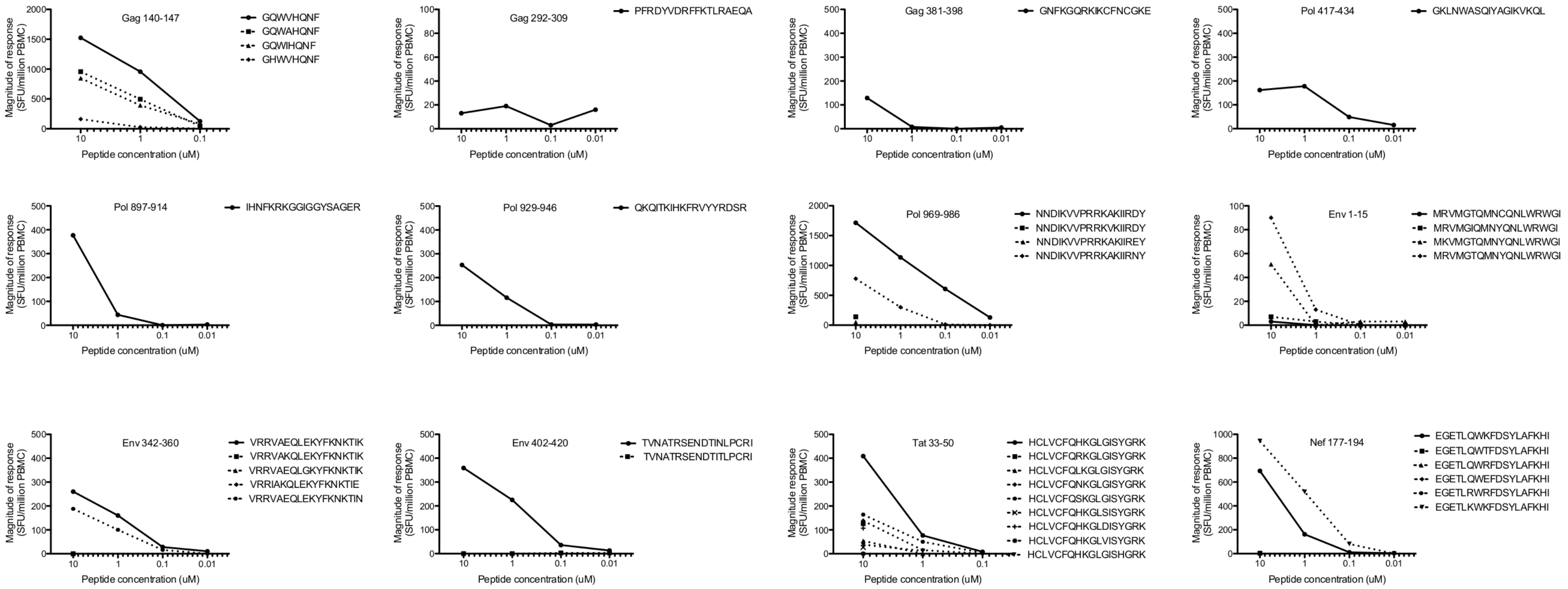
Magnitude of response (SFU/million PBMC)
Peptide concentration (uM)
Gag 140-147
GQWVHQNF
GQWAHQNF
GQWIHQNF
GHWVHQNF
Gag 292-309
PFRDYVDRFFKTLRAEQA
Gag 381-398
GNFKGQRKIKCFNCGKE
Pol 417-434
GKLNWASQIYAGIKVKQL
Pol 897-914
IHNFKRKGGIGGYSAGER
Pol 929-946
QKQITKIHKFRVYYRDSR
Pol 969-986
NNDIKVVPRRKAKIIRDY
NNDIKVVPRRKVKIIRDY
NNDIKVVPRRKAKIIREY
NNDIKVVPRRKAKIIRNY
Env 1-15
MRVMGTQMNCQNLWRWGI
MRVMGIQMNYQNLWRWGI
MKVMGTQMNYQNLWRWGI
MRVMGTQMNYQNLWRWGI
Env 342-360
VRRVAEQLEKYFKNKTIK
VRRVAKQLEKYFKNKTIK
VRRVAEQLGKYFKNKTIK
VRRIAKQLEKYFKNKTIE
VRRVAEQLEKYFKNKTIN
Env 402-420
TVNATRSENDTINLPCRI
TVNATRSENDTITLPCRI
Tat 33-50
HCLVCFQKKGLGISYGRK
HCLVCFQRKGLGISYGRK
HCLVCFQLKGLGISYGRK
HCLVCFQNKGLGISYGRK
HCLVCFQSKGLGISYGRK
HCLVCFQHKGLSISYGRK
HCLVCFQHKGLDISYGRK
HCLVCFQHKGLVISYGRK
HCLVCFQHKGLGISHGRK
Nef 177-194
EGETLQWKFDSYLAFKHI
EGETLQWTFDSYLAFKHI
EGETLQWRFDSYLAFKHI
EGETLQWEFDSYLAFKHI
EGETLRWRFDSYLAFKHI
EGETLKWKFDSYLAFKHI
