# Supplementary material for: Transmitted Virus Fitness and Host T Cell Responses Collectively Define Divergent Infection Outcomes in Two HIV-1 Recipients
Source: PLoS Pathog. 2015 Jan 8;11(1):e1004565. doi: 10.1371/journal.ppat.1004565 (PMC4287535; doi:10.1371/journal.ppat.1004565)
Supplement: S7 Fig — Amino acid alignments for variable regions in Env gp120 over the first year of infection for (A) R880F and (B) R463F. Sequences were compared to the T/F consensus sequence for each SC at the top of the alignments. Dots indicated that the residue was conserved, while dashes indicate a deletion/insertion. Amino acid substitutions from consensus are indicated. Gray stripes depict sites where mutations resulted in loss of a putative N-linked glycosylation motif (NxS/T); yellow stripes indicate the addition of putative N-linked glycosylation sites; cyan highlights in the consensus sequence identify stable N-linked glycosylation sites. (PDF) [file ppat.1004565.s007.pdf]

A.

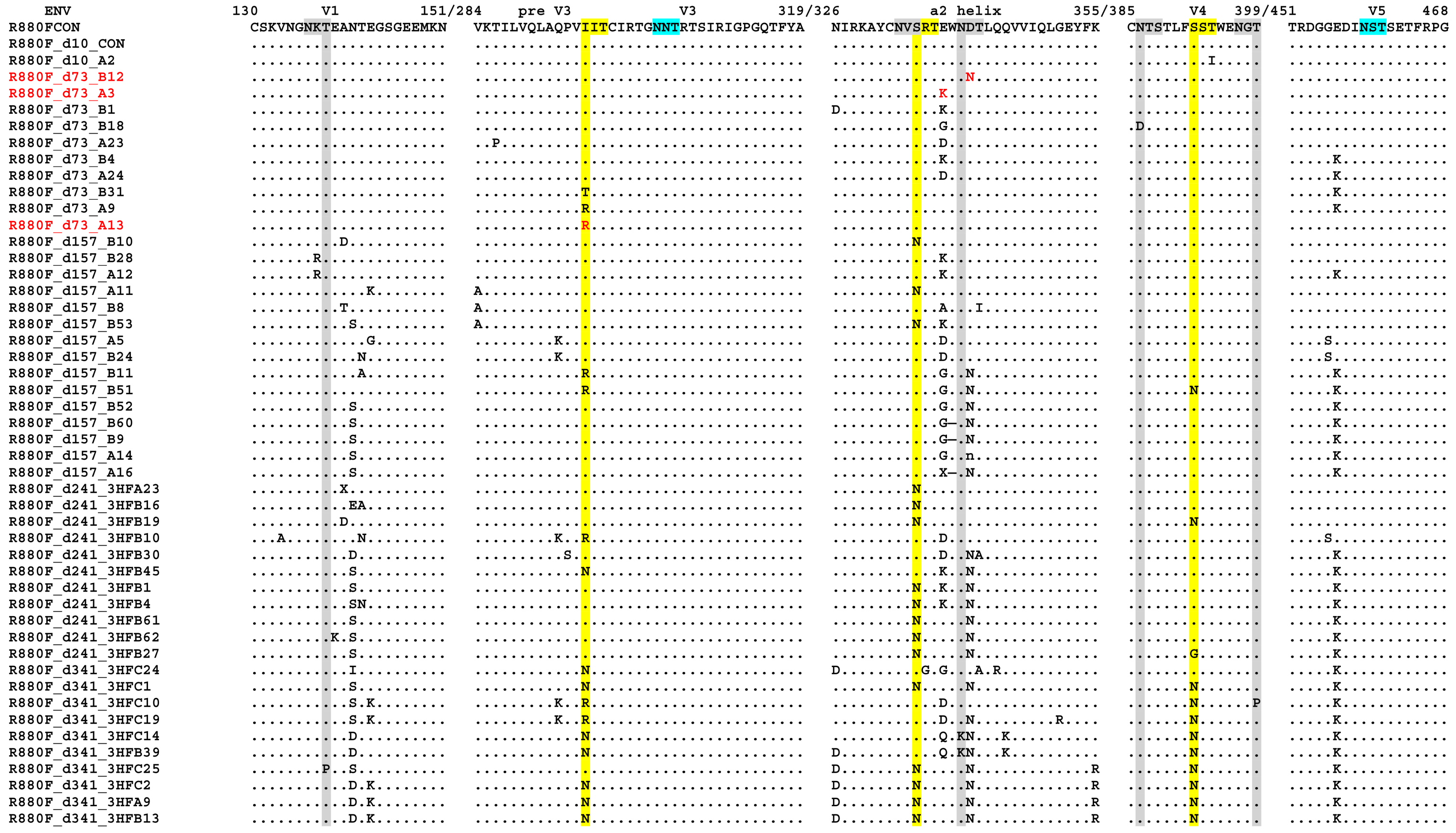
ENV
130
V1
151/284
pre V3
V3
319/326
a2 helix
355/385
V4
399/451
V5
468
R880FCON CSKVNGNKTEANTEGSGEEMKN VKTILVQLAQPVIITCIRTGNNTRTSIRIGPGQTFYA NIRKAYCNVSRTEWNDTLQQVVIQLGEYFK CNTSTLFSSTWENGT TRDGGEDINSTSETFRPG
R880F_d10_CON
R880F_d10_A2
R880F_d73_B12
R880F_d73_A3
R880F_d73_B1
R880F_d73_B18
R880F_d73_A23
R880F_d73_B4
R880F_d73_A24
R880F_d73_B31
R880F_d73_A9
R880F_d73_A13
R880F_d157_B10
R880F_d157_B28
R880F_d157_A12
R880F_d157_A11
R880F_d157_B8
R880F_d157_B53
R880F_d157_A5
R880F_d157_B24
R880F_d157_B11
R880F_d157_B51
R880F_d157_B52
R880F_d157_B60
R880F_d157_B9
R880F_d157_A14
R880F_d157_A16
R880F_d241_3HFA23
R880F_d241_3HFB16
R880F_d241_3HFB19
R880F_d241_3HFB10
R880F_d241_3HFB30
R880F_d241_3HFB45
R880F_d241_3HFB1
R880F_d241_3HFB4
R880F_d241_3HFB61
R880F_d241_3HFB62
R880F_d241_3HFB27
R880F_d341_3HFC24
R880F_d341_3HFC1
R880F_d341_3HFC10
R880F_d341_3HFC19
R880F_d341_3HFC14
R880F_d341_3HFB39
R880F_d341_3HFC25
R880F_d341_3HFC2
R880F_d341_3HFA9
R880F_d341_3HFB13

B.

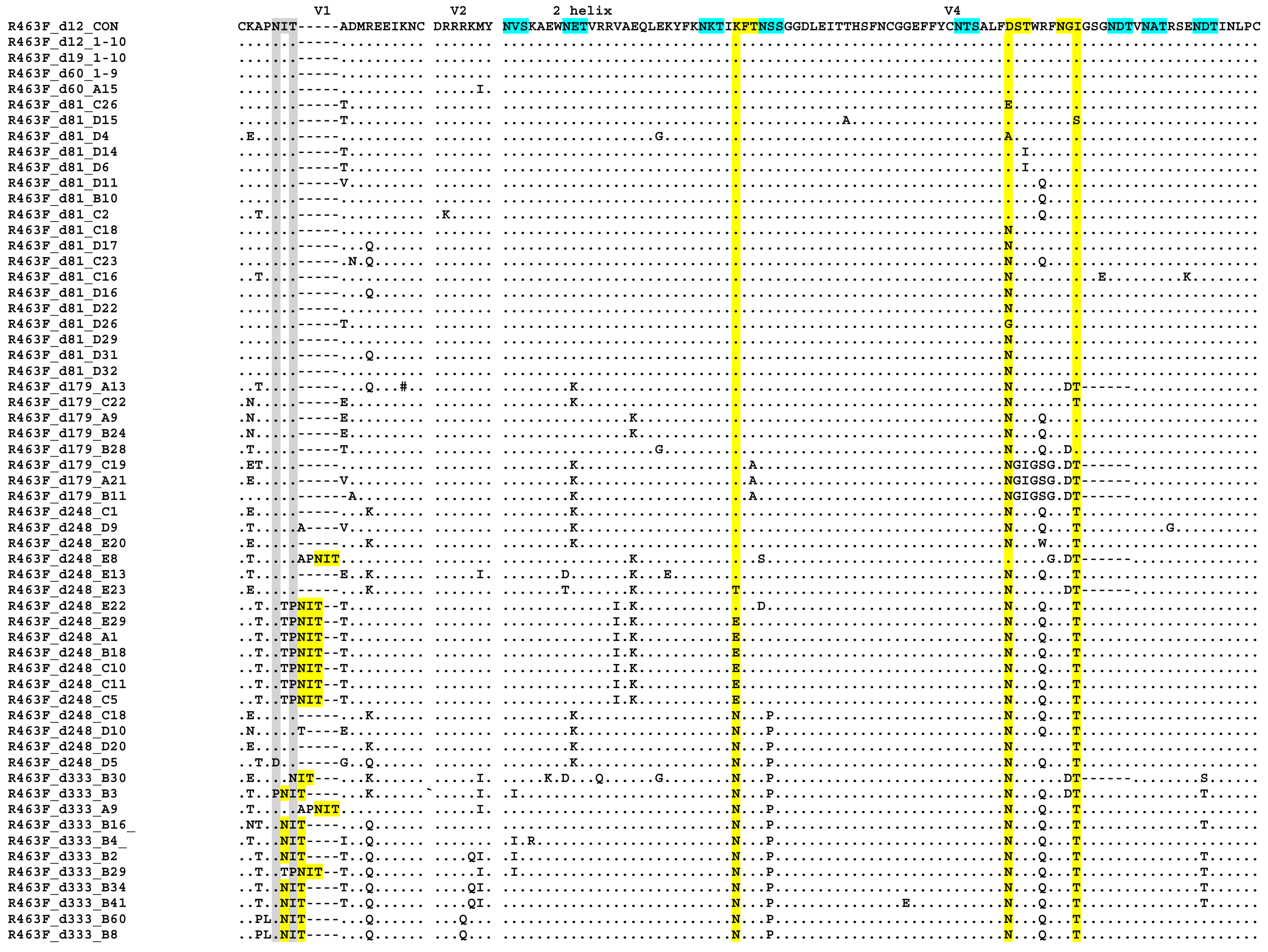
V1
V2
2 helix
V4
R463F_d12_CON CKAPNIT-----ADMREEIKNC DRRRKMY NVSKAEWNETVRRVAEQLEKYFKNKTIKFTNSSGGDLEITTHSFNCGGEFFYCNTSALFDSTWRFNGIGSGNDTVNATRSENDTINLPC
R463F_d12_1-10
R463F_d19_1-10
R463F_d60_1-9
R463F_d60_A15
R463F_d81_C26
R463F_d81_D15
R463F_d81_D4
R463F_d81_D14
R463F_d81_D6
R463F_d81_D11
R463F_d81_B10
R463F_d81_C2
R463F_d81_C18
R463F_d81_D17
R463F_d81_C23
R463F_d81_C16
R463F_d81_D16
R463F_d81_D22
R463F_d81_D26
R463F_d81_D29
R463F_d81_D31
R463F_d81_D32
R463F_d179_A13
R463F_d179_C22
R463F_d179_A9
R463F_d179_B24
R463F_d179_B28
R463F_d179_C19
R463F_d179_A21
R463F_d179_B11
R463F_d248_C1
R463F_d248_D9
R463F_d248_E20
R463F_d248_E8
R463F_d248_E13
R463F_d248_E23
R463F_d248_E22
R463F_d248_E29
R463F_d248_A1
R463F_d248_B18
R463F_d248_C10
R463F_d248_C11
R463F_d248_C5
R463F_d248_C18
R463F_d248_D10
R463F_d248_D20
R463F_d248_D5
R463F_d333_B30
R463F_d333_B3
R463F_d333_A9
R463F_d333_B16_
R463F_d333_B4_
R463F_d333_B2
R463F_d333_B29
R463F_d333_B34
R463F_d333_B41
R463F_d333_B60
R463F_d333_B8
