# Supplementary material for: Transmitted Virus Fitness and Host T Cell Responses Collectively Define Divergent Infection Outcomes in Two HIV-1 Recipients
Source: PLoS Pathog. 2015 Jan 8;11(1):e1004565. doi: 10.1371/journal.ppat.1004565 (PMC4287535; doi:10.1371/journal.ppat.1004565)
Supplement: S1 Table — Longitudinal sequence analysis of epitopes in virus isolated from individual R880F. (PDF) [file ppat.1004565.s008.pdf]

Table S1. Longitudinal sequence analysis of virus isolated from individual R880F

Epitope	Day12[a]	Day73[b]		Day157[b]		Day241[b]		Day323[b]	
Gag 17-34	EKIRLRPGGKKKYRMKHL	------------------	100%	------------------	100%	------------------	100%	------------------	100%
Gag 85-95	LYCVHQRIEVK	-----------<br>--W--------	90%<br>10%	-----------<br>-------M---	67%<br>33%	-----------	100%	-----------<br>-------M---	86%<br>14%
Gag 140-157	GQMIHQPLSPRTLNAWVK	------------------	100%	------------------	100%	------------------<br>------S-----------	83%<br>17%	------------------	100%
Pol 417-434	GKLNWASQIYPGIKVKQL	------------------	100%	------------------<br>-----------R------<br>--------------I---	33.3%<br>33.3%<br>33.3%	-------------Q----<br>--------------I---<br>---K---------QI---	66%<br>17%<br>17%	-------------Q----<br>------G------Q----<br>-------------QI---	72%<br>14%<br>14%
Pol 425-442	IYPGIKVKQLCKLLRGTK	------------------	100%	------------------<br>---R--------------<br>------I-----------	33.3%<br>33.3%<br>33.3%	-----Q------------<br>------I-----------<br>-----QI-----------	66%<br>17%<br>17%	-----Q------------<br>-----QI-----------	86%<br>14%
Pol 817-826	LKLAGRWPVK	----------	100%	----------	100%	----------	100%	----------	100%
Pol 969-986	NSDIKVVPRRKAKIIRDY	------------------<br>-----I------------	90%<br>10%	------------------	100%	------------------<br>-----I------------<br>--E--------V------	60%<br>20%<br>20%	------------------	100%
Pol 977-994	RRKAKIIRDYGKQMAGDD	------------------	100%	------------------	100%	------------------<br>---V--------------	80%<br>20%	------------------	100%
Pol 985-1002	DYGKQMAGDDCVAGRQDE	------------------	100%	------------------	100%	------------------	100%	------------------	100%
Env 350-368	GEYFKNKTITFNSSSGGD	------------------	100%	------------------	100%	------------------<br>----R-------------	91%<br>9%	----R-------------<br>------------------	60%<br>40%
Env 815-823	VDTIAIAVA	---------<br>------T--	80%<br>20%	-N-------<br>---------<br>-N----T--<br>-E-------<br>---T-----	46%<br>33%<br>7%<br>7%<br>7%	—N-------<br>---------<br>------T--<br>------V--	45%<br>27%<br>18%<br>9%	N--------<br>------T--	73%<br>27%
Rev 11-21	ELLRAIRIIKI	-----------	100%	---K-------<br>-----------	67%<br>33%	---K-------<br>-----------	82%<br>18%	---K-------	100%
Vif 25-42	VKHHMYVSKRAKRWFYRH	------------------	100%	------------------<br>---Q--------------	67%<br>33%	------------------<br>-----H------------<br>------A-----------	75%<br>17%<br>8%	--------R---------<br>-----H------------<br>------I-----------<br>------------------	64%<br>18%<br>9%<br>9%
Nef 177-194	EREVLKWKFDSRLALKHL	-------R----------<br>----------C-------	90%<br>10%	-------R----------	100%	-------R----------<br>----------CH-F----	91%<br>9%	-------R----------<br>-------R-----F----	56%<br>44%

[a] Epitope Amino acid sequence in the autologous virus at the earliest timepoint available for analysis (10 days post-Fiebig I/II)
[b] The proportion of viral genomes sequenced that retained the founder virus epitope sequence (indicated by dashes) or the mutant versions thereof at the indicated time-points (days post-Fiebig I/II)
