# Supplementary material for: Transmitted Virus Fitness and Host T Cell Responses Collectively Define Divergent Infection Outcomes in Two HIV-1 Recipients
Source: PLoS Pathog. 2015 Jan 8;11(1):e1004565. doi: 10.1371/journal.ppat.1004565 (PMC4287535; doi:10.1371/journal.ppat.1004565)
Supplement: S2 Table — Longitudinal sequence analysis of epitopes in virus isolated from individual R463F. (PDF) [file ppat.1004565.s009.pdf]

Table S2 - Longitudinal sequence analysis of virus isolated from individual R463F

Epitope	Day 12[a]	Day 19[b]	Day 60[b]	Day 179[b]	Day 248[b]	Day 333[b]
Gag 132-149	YPVVQNAQGQWVHQNFSP	------------------ 100%	------------------ 100%	-----------A------ 87.5%<br>-----------I------ 12.5%	-----------A------ 37.5%<br>---------H-------- 37.5%<br>------------P----- 12.5%<br>--------------H--- 12.5%	-----------A------ 44%<br>---------H-------- 33%<br>-----------I------ 11%<br>-----------T------ 11%
Gag 140-157	GQWVHQNFSPRTLNAWVK	------------------ 100%	------------------ 100%	---A-------------- 87.5%<br>---I-------------- 12.5%	---A-------------- 37.5%<br>-H---------------- 37.5%<br>----P------------- 12.5%<br>------H----------- 12.5%	---A-------------- 44%<br>-H---------------- 33%<br>---I-------------- 11%<br>---T-------------- 11%
Gag 292-309	PFRDYVDRFFKTLRAEQA	------------------ 100%	------------------ 100%	------------------ 100%	------------------ 100%	------------------ 100%
Gag 381-398	GNFKGQRKIKCFNCGKE	------------------ 100%	------------------ 100%	------------------ 100%	------------------ 100%	------------------ 100%
Pol 409-426	VNDIQKLVGKLNWASQIY	------------------ 100%	------------------ 100%	------------------ 100%	------------------ 100%	------------------ 100%
Pol 417-434	GKLNWASQIYAGIKVKQL	------------------ 100%	------------------ 100%	------------------ 100%	------------------ 100%	------------------ 100%
Pol 897-914	IHNFKRKGGIGGYSAGER	------------------ 100%	------------------ 100%	------------------ 100%	------------------ 100%	------------------ 100%
Pol 929-946	QKQITKIHKFRVYYRDSR	------------------ 100%	------------------ 100%	------------------ 100%	------------------ 100%	------------------ 100%
Pol 969-986	NNDIKVVPRRKAKIIRDY	------------------ 100%	------------------ 100%	------------------ 75%<br>-----------V------ 12.5%<br>----------------E- 12.5%	------------------ 87.5%<br>--------X--------- 12.5%	------------------ 55%<br>-----------V------ 22%<br>K----------V------ 11%<br>----------------N- 11%
Pol 977-994	RRKAKIIRDYGKQMAGD	------------------ 100%	------------------ 100%	----------------- 75%<br>---V------------- 12.5%<br>--------E-------- 12.5%	----------------- 87.5%<br>X---------------- 12.5%	----------------- 55%<br>---V------------- 33%<br>--------N-------- 11%
Pol 985-1002	DYGKQMAGDDCVAGRQDE	------------------ 100%	------------------ 87.5%<br>--------------G--- 12.5%	------------------ 87.5%<br>E----------------- 12.5%	------------------ 100%	------------------ 78%<br>N----------------- 11%<br>----------M------- 11%
Env 1-15	MRVMGTQMNYQNLWRWGI	------------------ 100%	------------------ 100%	------------------ 63%<br>---------C-------- 25%<br>-----I------------ 12%	------------------ 50%<br>-K---------------- 25%<br>---------C-------- 12.5%<br>---T-------------- 12.5%	-K---------------- 78%<br>---------C-------- 22%
Env 334-351	SKAEWNETVRRVAEQLEK	------------------ 100%	------------------ 100%	------------------ 67%<br>-----K------------ 22%<br>--------G--------- 11%	-----------------N 75%<br>---I-K-----------E 25%	-----------------N 91%<br>-Q------G--------N 9%
Env342-360	VRRVAEQLEKYFKNKTIK	------------------ 100%	------------------ 100%	------------------ 62.5%<br>-----K------------ 25%<br>--------G--------- 12.5%	-----------------N 50%<br>---I-K-----------E 37.5%<br>------------------ 12.5%	-----------------N 89%<br>—Q------G--------N 11%
Env 402-420	TVNATRSENDTINLPCRI	------------------ 100%	------------------ 100%	------------------ 100.0%	------------------ 100%	------------------ 44.5%<br>------------T----- 44.5%<br>------------S----- 11%
Tat 33-50	HCLVCFQHKGLGISYGRK 90%<br>-----------D------ 10%	-------R---------- 36%<br>-----------S------ 21%<br>------------------ 14%<br>-----------D------ 7%<br>-----------V------ 7'%<br>--------------H--- 7%<br>-------R------H--- 7%	-------S---------- 62.5%<br>--------------H--- 12.5%<br>-------L---------- 12.5%<br>-------N---------- 12.5%	-------N---------- 37.5%<br>-------L---------- 37.5%<br>-------S---------- 25%	-------N---------- 75%<br>-------L---------- 25%	-------N---------- 45%<br>-------L---------- 33%<br>-------S---------- 22%
Nef 177-194	EGETLQWKFDSYLAFKHI	------------------ 100%	------------------ 37.5%<br>-------E---------- 37.5%<br>-------T---------- 12.5%<br>-------R---------- 12.5%	-------R---------- 87.5%<br>-------T---------- 12.5%	-------R---------- 37.5%<br>-----R-R---------- 37.5%<br>-----K------------ 25%	-----R-R---------- 100%

[a] Epitope Amino acid sequence in the autologous virus at the earliest time-point available for analysis (3 days post Fiebig I/II)
[b] The proportion of viral genomes sequenced that retained the founder virus epitope sequence (indicated by dashes) or the mutant versions thereof at the indicated time-points (days post-Fiebig I/II)
